# Patient–ventilator synchrony under non-invasive ventilation is improved by an automated real time waveform analysis algorithm: a bench study

**DOI:** 10.1186/s40635-025-00726-y

**Published:** 2025-02-12

**Authors:** Yann Renaud, Jocelyne Auroi, Davy Cabrio, Ermes Lupieri, Jean-Daniel Chiche, Lise Piquilloud

**Affiliations:** 1 Adult Intensive Care Unit, Lausanne University Hospital, Lausanne, Switzerland; 2 Faculty of Biology and Medicine, University of Lausanne, Lausanne, Switzerland; 3 Orthopedics & Traumatology of the Musculoskeletal System, Bürgerspital, Solothurn, Solothurn, Switzerland

**Keywords:** Non-invasive ventilation, NIV, Patient–ventilator synchrony, Asynchrony, Waveform analysis, Bench study

## Abstract

**Background:**

Because of inherent leaks, obtaining good patient–ventilator synchrony during non-invasive ventilation (NIV) is challenging. The IntelliSync + ^®^ software (Hamilton medical, Bonaduz, CH), that can be used together with the NIV mode, performs real-time automated analysis of airway pressure- and flow-time curves to detect the transition between inspiration and expiration. It then controls the ventilator inspiratory and expiratory valves to improve patient–ventilator synchrony. The main goal of this NIV bench study was to evaluate the impact of IntelliSync + ^®^ on synchrony in the presence of leaks of 9 and 20 L/min in the tested ventilator circuit (no face mask used), with normal, obstructive and restrictive respiratory mechanics and two levels of NIV pressure support (PS 8 and 14 cmH_2_O). For this, the time needed to trigger the ventilator (Td) and the difference between the end of the simulated breath and the termination of pressurization (Tiex) were measured. The number of classical asynchronies and the ventilator pressurization capacity were also assessed.

**Results:**

Compared to NIV delivered with the classical NIV mode (compensating leaks and limiting inspiratory time to 2 s), activating IntelliSync + ^®^ improved Tiex and, to a lesser extent, Td in clinically relevant setups. IntelliSync + ^®^ also showed a trend towards reducing classical asynchronies, particularly directly after leak flow increase. The impact of the system was most significant with high PS levels and pathological respiratory mechanics. Especially, in the obstructive model, in the presence of large leak (20 L/min) and PS 14 cmH_2_O, Tiex decreased from 0.61 [0.56–0.64] to 0.16 [0.07–0.18] s and Td from 0.07 [0.06–0.08] to 0.06 [0.06–0.08] s. In less challenging situations, IntelliSync + ^®^ was less beneficial. Overall, ventilator pressurization was improved when IntelliSync + ^®^ was activated.

**Conclusions:**

In this NIV bench model, IntelliSync + ^®^, used in addition to NIV-PS, improved both expiratory and inspiratory synchrony. It was particularly efficient in the presence of obstructive and restrictive respiratory mechanics and high-pressure support levels. These pre-clinical results tend to support the ability of IntelliSync + ^®^ to improve patient–ventilator synchrony in the presence of leaks and provide pre-clinical data supporting a clinical evaluation of the automated algorithm during NIV.

**Supplementary Information:**

The online version contains supplementary material available at 10.1186/s40635-025-00726-y.

## Background

Poor patient–ventilator synchrony, defined as a mismatch between neural inspiratory effort and ventilator delivered pressurization [1], has been associated with altered patient comfort and worse outcome [2–5]. It may be related to patient characteristics (e.g., abnormal respiratory mechanics or weak inspiratory efforts) and/or ventilator performance and settings. In addition, during non-invasive ventilation (NIV), the presence of unavoidable leaks at the interface favors asynchronies by altering the flow-time curve. In fact, severe asynchrony, has been reported in up to 43% of the patients during pressure support NIV (NIV-PS) [6]. Activating “NIV mode” on ICU ventilators that partially compensates for leaks and often limits the maximal inspiratory time of the ventilator, overall improves patient–ventilator synchrony. It, however, does not completely alleviate the asynchronous breaths [7]. Recent advancements in automation and real-time analysis of airway pressure- and flow-time curves led to the development of the IntelliSync + ^®^ software (Hamilton Medical, Bonaduz, Switzerland), that aims to further improve patient–ventilator synchrony by automatically detecting changes in shape in the flow and airway-pressure time curves related to the transition between active inspiration and passive expiration. In practice, a short period of passive inflation enables the detection of the end of active inspiration. On the flow-time curve, this corresponds to the transition from a fast to a slow exponential decay of inspiratory flow [8]. The IntelliSync + ^®^ software can be activated on the Hamilton S1 ventilator screen using a dedicated button. The system can be activated by the clinician either during both the inspiratory and expiratory phases (full activation) or during one of these 2 phases only (partial activation of the system). When IntelliSync + ^®^ is fully activated, the algorithm controls the inspiratory and expiratory ventilator valves aiming to optimize the matching between the patient’s breath and the ventilator delivered pressurization. When IntelliSync + ^®^ is partially activated, only one of the two ventilator valves is automatically controlled by the algorithm. In a recent clinical study, IntelliSync + ^®^ improved inspiratory and expiratory synchrony in difficult to wean invasively ventilated patients [9]. The IntelliSync + ^®^ algorithm can also be used in conjunction with the NIV mode. However, its performance in this setting and in the presence of leaks has not yet been studied. This raises potential concerns, as leaks can affect airway pressure- and flow-time curves, which the automated system relies on to optimize synchrony. Assessing the performance of IntelliSync + ^®^ in the presence of leaks in an experimental setting is desirable before testing it in non-invasively ventilated patients.

The main aim of this study was to evaluate whether the IntelliSync + ^®^ software, when activated together with the NIV-mode, could improve patient–ventilator synchrony in a NIV model simulating different levels of leaks, and normal and pathologic respiratory mechanics. As secondary aim, we assessed the effect on synchrony of only partially activating IntelliSync + ^®^, in comparison with its full activation. Finally, as third aim, we assessed the ventilator pressurization capacity with and without the IntelliSync + ^®^ software activated.

## Methods

### Bench description

**Fig. 1:**
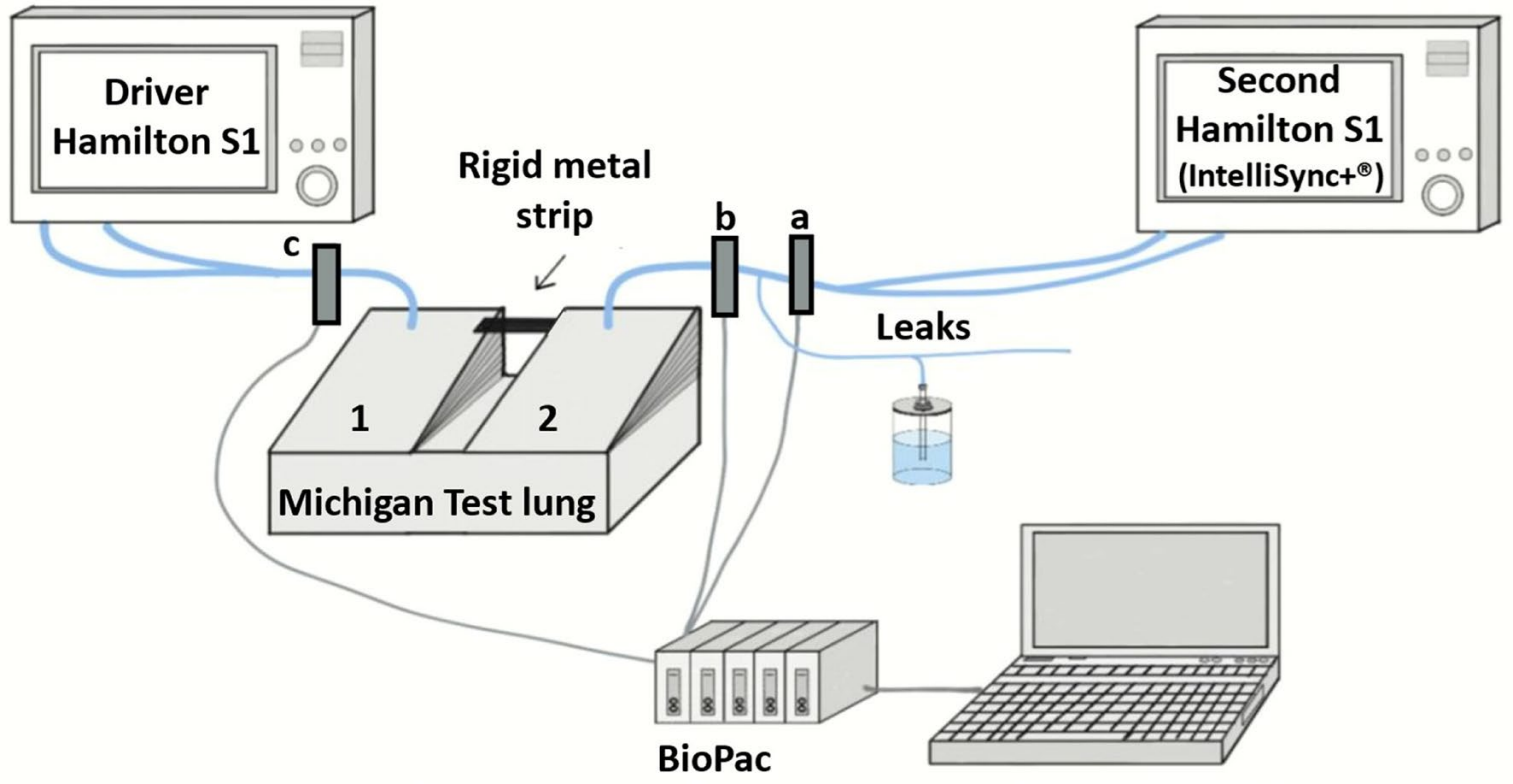
Bench test setting. Diagram illustrating the bench test setting. Compartment 1 represents the compartment connected to the driver ventilator, while compartment 2 represents the compartment connected to the second ventilator equipped with the IntelliSync + ^®^ software

Figure 1 depicts the bench model setup, which was made of a two-compartment Michigan test-lung (Michigan Instruments, Grand Rapids, USA) and two Hamilton-S1 ventilators (Hamilton Medical, Bonaduz, Switzerland). The test lung first compartment was connected to a first Hamilton-S1 driver ventilator used to simulate an inspiratory effort of moderate intensity (airway occlusion pressure at 100 ms (P0.1) of − 5.58 ± 0.29 cmH_2_O and global occlusion pressure (P_occl_) of − 13.48 ± 0.90 cmH_2_O) [10, 11]. Briefly, in response to each pressurization delivered by the driver ventilator, the test lung first compartment was inflated. A rigid metal strip attached to it simultaneously lifted the edge of the second compartment leading to a depression inside it, simulating an inspiratory effort (law of Boyle-Mariotte), that could be detected by the second Hamilton-S1 ventilator connected to this second compartment (using a standard non-humidified double-limb ventilator circuit). No facemask was used to simulate the NIV interface, but leaks were created in the second ventilator circuit as close as possible to the test lung. The driver ventilator was set in a pressure regulated mode as previously described [1]. In practice, we used Airway Pressure Release Ventilation mode (APRV) as with this mode it is easy to set inspiratory and expiratory time and to select the level of assist. The detailed settings are mentioned in Table 1. The second Hamilton ventilator, equipped with the IntelliSync + ^®^ software, was set in NIV-PS. On the Hamilton-S1 ventilator, the NIV-PS mode, in addition to compensating for leaks, comes with a by default maximum inspiratory time of 2 s, that was not modified. For the tests, two levels of pressure support (PS) of 8 or 14 cmH_2_O were used to simulate low and high level of assist, respectively. These two levels where chosen arbitrarily to be clinically relevant and in line with previously published bench models [12, 13]. Using the dedicated button on the ventilator screen, IntelliSync + ^®^ was sequentially deactivated (IS0), activated during inspiration (ISI), during expiration (ISE) and finally during both the inspiratory and expiratory phases (full activation, ISIE). In the absence of IntelliSync + ^®^ activation on the corresponding phase of the breathing cycle, the inspiratory and expiratory triggers thresholds were set according to usual clinical practice based on expert opinion. For inspiratory trigger, a value of 1.5 L/minute was selected. This is in line with the strategy used by Carteaux et al. in their NIV bench test, who used the most sensible setting while avoiding auto-triggering [14]. For expiratory trigger, the following settings were chosen: high expiratory trigger threshold and short pressurization time for obstructive respiratory mechanics, and low expiratory trigger threshold and short pressurization time for restrictive mechanics. The detailed settings used for the second ventilator are mentioned in Table 1.

**Table 1:** Ventilator settings, and compliance and resistance values used to simulate the different respiratory mechanics

	Normal respiratory mechanics	Obstructive respiratory mechanics	Restrictive respiratory mechanics
**Driver ventilator settings**			
Ventilation mode	Assisted pressure release ventilation (APRV)		
High pressure time [s]	0.9	0.9	0.8
High pressure [cmH_2_O]	28	28	40
Low pressure time [s]	2.4	3.4	2.4
Low pressure [cmH_2_O]	9	10	16
Respiratory rate [cycles/min]	18^a^	14^a^	18^a^
Pressurization slope [ms]	200		
FiO_2_ [%]	21		
**Second ventilator settings**			
Ventilation mode	Pressure support (NIV mode)		
Pressure support level [cmH_2_O]	8 or 14		
Maximum inspiratory time [s]	2		
Inspiratory trigger threshold [L/min]	1.5^b^		
Pressurization slope [ms]	150	100	100
Expiratory trigger setting [%]	25^c^	40^c^	15^c^
Maximum inspiratory time [s]	2		
FiO_2_ [%]	21		
PEEP [cmH_2_O]	5		
**Respiratory mechanics parameters**			
Compliance [ml/cmH_2_O]	78.3 ± 1.7	81.4 ± 1.7	49.4 ± 0.5
Resistance [cmH_2_O/L/s]	7.0 ± 0.3	24.6 ± 0.7	7.0 ± 0.2

^a^ Respiratory rate was set at 18/min, or, for obstructive respiratory mechanics, at the highest possible rate without generating auto-PEEP in the absence of leak

^b^When IntelliSync + ^®^ is deactivated during inspiration

^c^When IntelliSync + ^®^ is deactivated during expiration

As no face mask was used in this bench model, calibrated leaks were created in the second ventilator circuit by placing a 22–22 male–female connector with an open lateral port (Intersurgical, East Syracuse, NY, USA) as close as possible to the second compartment of the test lung. This strategy aimed at stimulating leaks at the NIV interface. As previously described in another NIV bench-test, two different types of leaks were sequentially created for each tested condition [14]: inspiro-expiratory leaks and leaks limited to inspiration to, respectively, evaluate inspiratory (triggering) and expiratory (cycling-off) synchrony [14]. Two different leak levels were created for each type of leak, a small leak (L9, target 9 L/min) and a large leak (L20, target 20 L/min) [14]. The condition without leak was named L0, (target 0 L/min). The leak flow levels used were arbitrary. They were selected because of clinical relevance and because they were in line with the leak levels used in other NIV bench studies [1, 14]. A detailed description of the system used to create the leaks and to measure the effective leaks obtained is mentioned in the *Electronic Supplementary Material* (*ESM*), *eFigures 1–2*, as are the leak values measured (*eTables 1–2*).

**Fig. 2:**
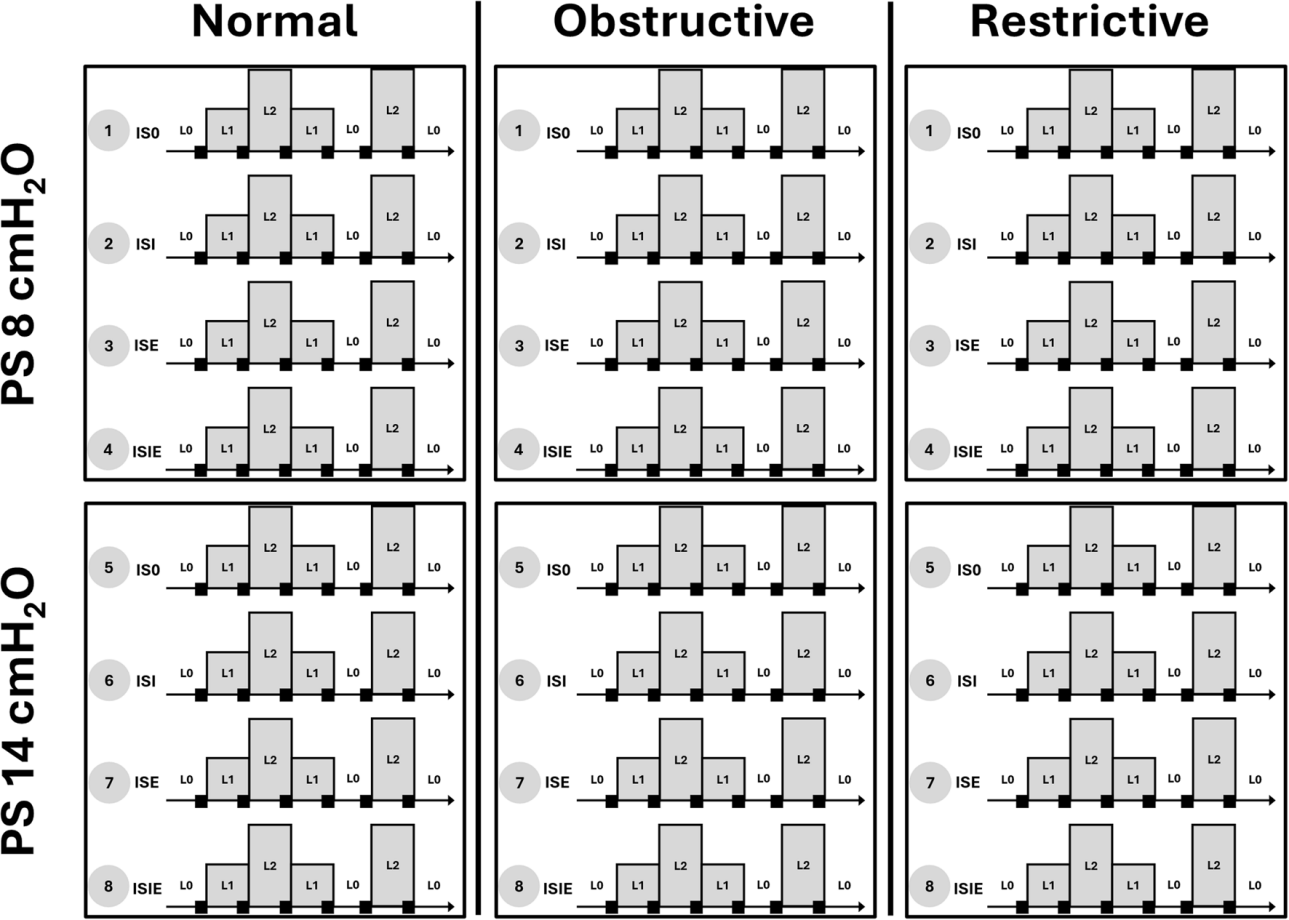
Description of the data acquiring. The data was collected sequentially for every IntelliSync + ^®^ activation mode, with the different pressure support levels (8 and 14 cmH_2_O) and the three respiratory mechanics (normal, obstructive and restrictive). Black box represents the analyzed data. L0: no leak (0 L/min); L1: small leak flow (9 L/min); L2: high leak flow (20 L/min); IS0: IntelliSync + ^®^ deactivated; ISI: IntelliSync + ^®^ activated during inspiration only; ISE: IntelliSync + ^®^ activated during expiration only; ISIE: IntelliSync + ^®^ activated during inspiration and expiration

The performance of the IntelliSync + ^®^ algorithm was sequentially and independently assessed with normal, obstructive, and restrictive respiratory mechanics, and with sequentially a pressure support of 8 and 14 cmH_2_O (Fig. 2). Resistance and compliance parameters used for each respiratory mechanic are mentioned in Table 1 and details are available in the *ESM.* They are in line with values that can be measured in patients undergoing NIV in clinical practice and with values previously used in other bench tests [1, 11, 12].

### Data recording and analysis

An analogic-to-digital converter Biopac 100^®^ was connected to the flow and pressure sensors placed in the ventilatory circuits (Fig. 1) and to a laptop computer equipped with Acqknowledge version 3.9.1 (Biopac System Inc, Goleta CA, USA). It enabled the recording of the flow and pressure–time curves at a sampling frequency of 50 Hz (maximal resolution of 20 ms). For each condition tested, a 5-min stabilization period without settings or leak modifications took place before starting the recording. Fifteen baseline ventilatory cycles without leak were first recorded. Then, different leak flows were generated in the following sequence: L0 to L9 to L20 to L9 to L0 to L20 to L0. The leak flow was modified every 2 min, to enable system stabilization at each step. Data was collected sequentially and independently for each respiratory mechanics and pressure support levels. Each IntelliSync + ^®^ activation modality (IS0, ISI, ISE, ISIE) was sequentially applied for each condition before starting with a new one (Fig. 2).

**Fig. 3:**
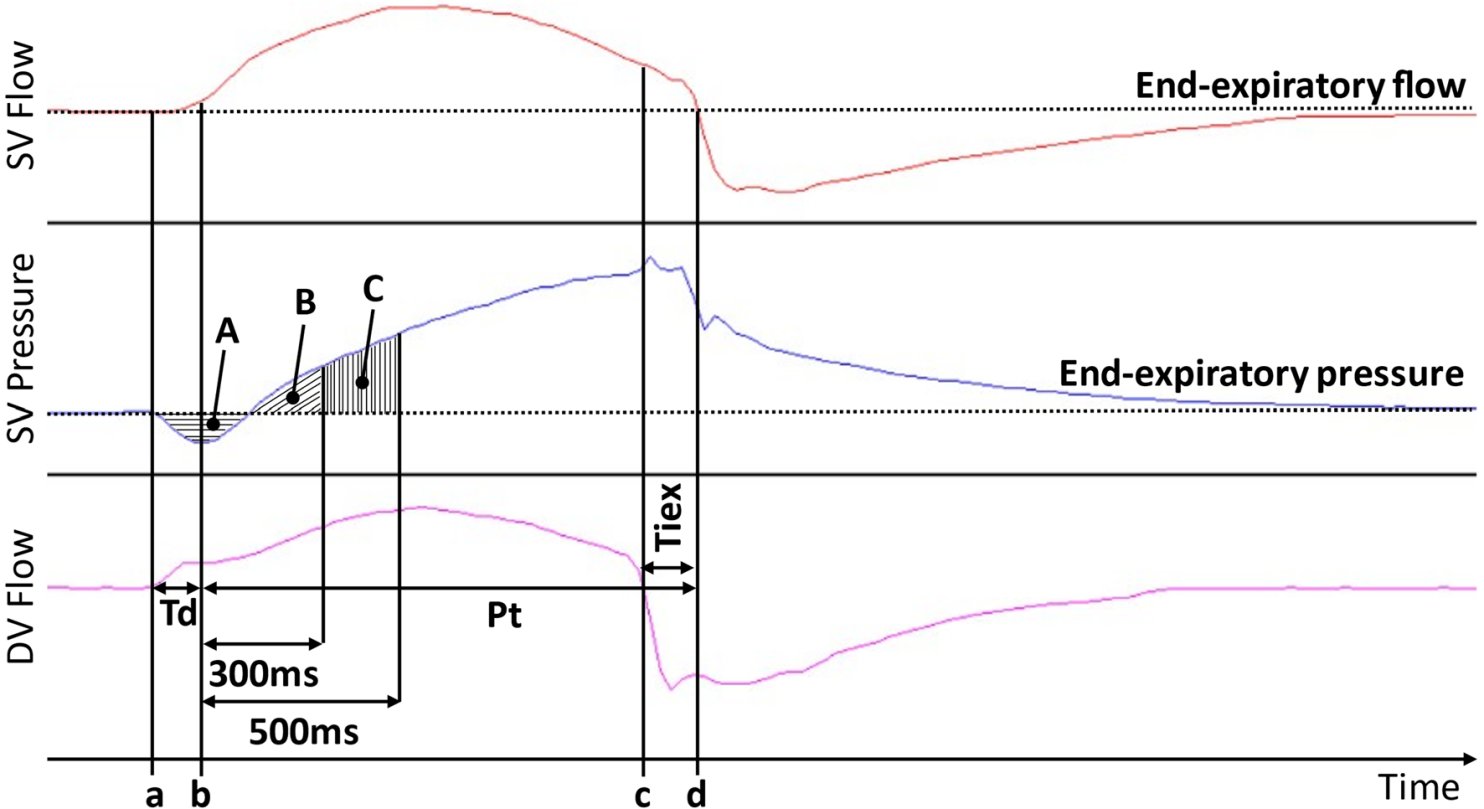
Time delays and pressure measurements definitions. *Td* trigger delay, *Pt* pressurization time, *Tiex* inspiratory time in excess, *SV* second ventilator, *DV* driver ventilator. Dashed lines represent end-expiratory flow, respectively, end-expiratory pressure. **a** Initial pressure drop corresponding to the start of the inspiratory effort. **b** Nadir of pressure corresponding to the start of pressurization. **c** End of inspiratory effort. **d** Return to end-expiratory flow of the second ventilator corresponding to the end of pressurization. PTP_vent_ trigger is defined as area A. PTP_vent_300 is defined as area B-|A| and PTP_vent_500 is defined as area B + C-|A|

The recorded curves were analyzed in Acqknowledge version 3.9.1. Synchrony was assessed according to previous publications through the measurements of trigger delay (Td) and inspiratory time in excess (Tiex) [1, 9, 14–16]. Td (Fig. 3) was defined as the time between the onset of the inspiratory effort (initial pressurization of the driver ventilator) and the start of pressurization delivered by the second ventilator (nadir of airway pressure) [10]. Tiex (Fig. 3) corresponded to the time between the end of the driver ventilator simulated breath (simulating the end of neural inspiratory effort) and the return to “baseline” flow of the second ventilator. Tiex could be negative (end of pressurization delivered by the second ventilator occurred before the end of simulated neural inspiratory effort, corresponding to the concept of premature cycling) or positive (end of pressurization delivered by the second ventilator occurred after the end of simulated neural inspiratory effort, corresponding to the concept of delayed cycling) (*eFigure 3* in the *ESM*). To assess pressurization capacity, the ventilator pressure–time product of the triggering phase (PTP_vent_trig) and the ventilator pressure–time product at 300 and 500 ms (PTP_vent_300 and PTP_vent_500) were used (Fig. 3) [17]. Details on their measurements are available in the *ESM*.

All numeric measurements related to synchrony and pressurization capacity were performed at steady state (i.e., on the ventilatory cycles recorded immediately before each subsequent leak modification) on the curves recorded by the flow and pressure sensor located between the second ventilator and the leakage system (sensor (a), Fig. 1). In practice, when the five cycles recorded immediately before a leak modification were all triggered by the driver ventilator, they were used for the measurements. If one or more of these cycles were not triggered, previous cycles were considered to find a total of 5 consecutive triggered cycles. If this did not occur during the 14 cycles recorded immediately before a leak change, the system was deemed unstable, and no measurements were taken.

The classical asynchronies previously defined by Thille et al. [18] (Table 2)**,** were counted during a span of 10 driver ventilator cycles, immediately before (steady state) or after (post-modification) each change of leak flow by examining the recorded pressure- and flow-time curves. Results are expressed as a percentage, using the *asynchrony index* (AI). For each classical asynchrony, the AI was calculated as the number of events divided by the total respiratory rate, computed as the sum of all triggered ventilatory cycles plus ineffective efforts [18].

**Table 2:** Definitions of classical asynchronies

Classical asynchrony	Definition
Auto-triggering	Ventilatory cycle by the second ventilator occurs despite the absence of a positive flow in the driver ventilator circuit
Ineffective effort	Absence of ventilatory cycle by the second ventilator, despite the presence of a positive flow in the driver ventilator circuit
Double-triggering	Succession of two ventilatory cycles delivered by the second ventilator with a short expiratory time between the cycles (< 50% of the mean inspiratory time). Only the first cycle is caused by a positive flow in the driver ventilator circuit
Late cycling	Insufflation time of the second ventilator is > 2 × the mean neural inspiratory time
Early cycling	Insufflation time of the second ventilator is < ½ x mean neural inspiratory time

In our bench model, the mean neural inspiratory time corresponds to the mean time of the driver *ventilator pressurization phase*

### Statistical analysis

Results are expressed as medians and interquartile range. Due to the relatively low sample size, the distribution was not tested, and non-parametric tests were used. Continuous data, including all timings delays and pressurization indexes, were compared between full and no activation of IntelliSync + ^®^ independently for each experimental setting (respiratory mechanics and level of pressure support) and each leak level (L9 or L20) using Wilcoxon rank-sum tests. Wilcoxon rank-sum tests were also used to, respectively, compare for each experimental setting Td between ISIE and ISI and Tiex between ISIE and ISE. The number of classical asynchronies was compared between full and no activation of IntelliSync + ^®^ for each experimental setting using the same approach and statistical test.

*P* value < 0.05 was considered as statistically significant. IBM^®^ SPSS^®^ Statistics version 29.0.1.0 (IBM, Armonk, USA) was used for statistical analysis.

## Results

All the 36 planned setups (3 respiratory mechanics, 2 pressure support levels, 3 leak conditions and IntelliSync + ^®^ deactivated or fully activated) could be recorded. Three setups (8.3%) were unstable, precluding the measurement of timings and pressurization parameters. All these three unstable setups occurred when continuous leaks were present with restrictive respiratory mechanics (L9 and PS 8 cmH_2_O without IntelliSync + ^®^ activated and L20 and PS 8 cmH_2_O without and with IntelliSync + ^®^ activated).

### Comparison of synchrony between IntelliSync + ^®^ deactivation and IntelliSync + ^®^ full activation (IS0 vs ISIE)

All detailed results are available in the *ESM*, *eTables 3–8*.

#### Trigger delay

**Fig. 4:**
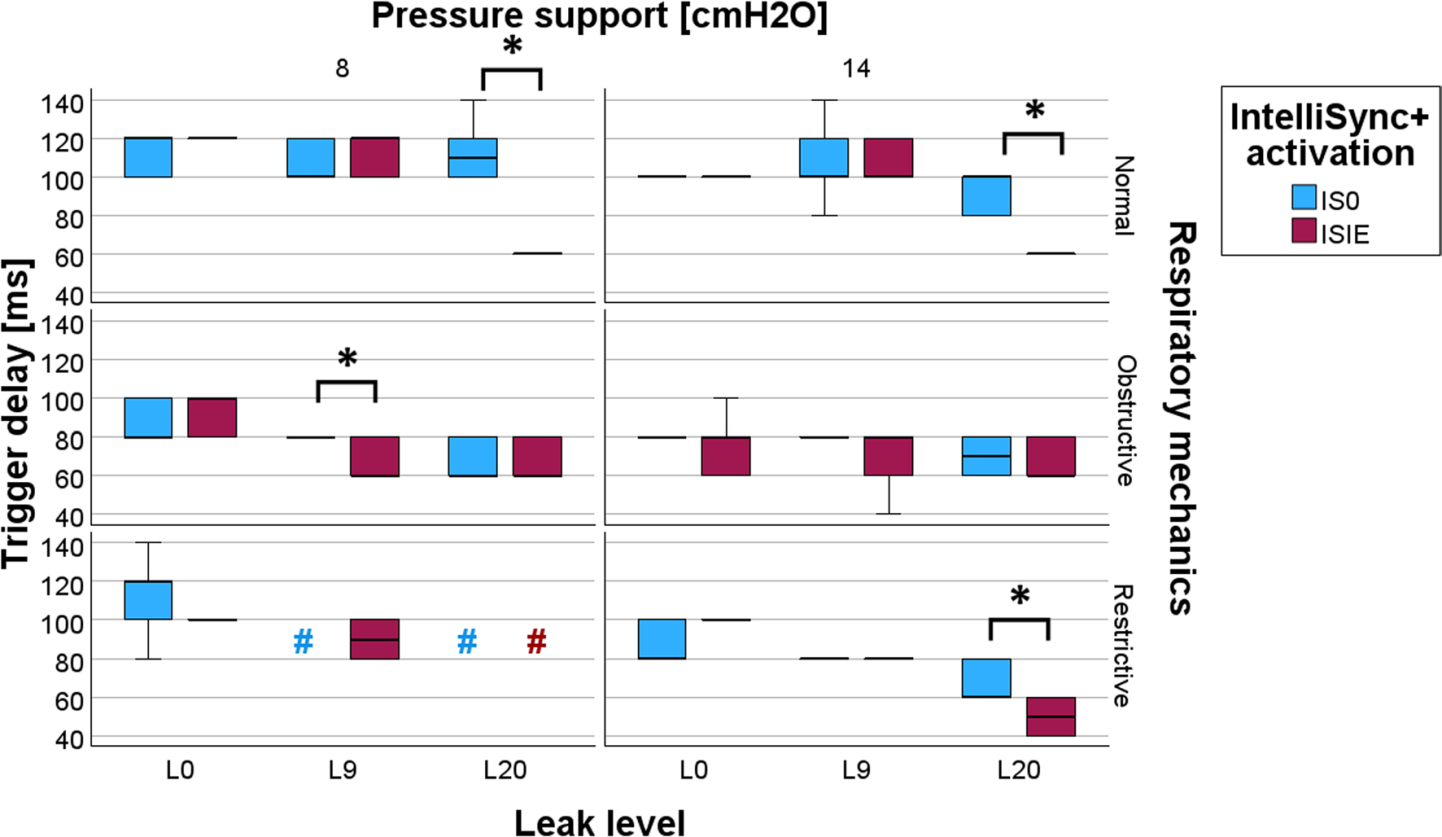
Trigger delays with and without IntelliSync + ^®^ fully activated in the tested setups. Borders of the colored boxes represent the first and third quartile. The thick line in the middle represents the median. Finally, the lower and upper whisker represent the 5th and 95th percentile, respectively. *p < 0.05, in favor of ISIE. *L0* no leak flow, *L9* leak flow of 9L/min, *L20* leak flow of 20 L/min, *IS0* IntelliSync + ^®^ deactivated, *ISIE* IntelliSync + ^®^ fully activated during both inspiratory and expiratory phases. # Unstable setup, with no trigger delay measurement possible

The Td measured with and without the fully activated IntelliSync + ^®^ (ISIE) are shown in Fig. 4 for the different experimental conditions. The precise numeric values of Td are provided in the *ESM*. In the presence of leaks, ISIE reduced Td in four different situations. First, with a small leak (L9), Td was reduced by ISIE in presence of obstructive respiratory mechanics and low PS level (8 cmH_2_O). In the presence of a large leak (L20), ISIE improved Td in normal respiratory mechanics with both PS levels (8 or 14 cmH_2_O), and in restrictive respiratory mechanics with high PS (14 cmH_2_O). In the absence of leak (L0), no significant differences on Td were found.

#### Inspiratory time in excess

**Fig. 5:**
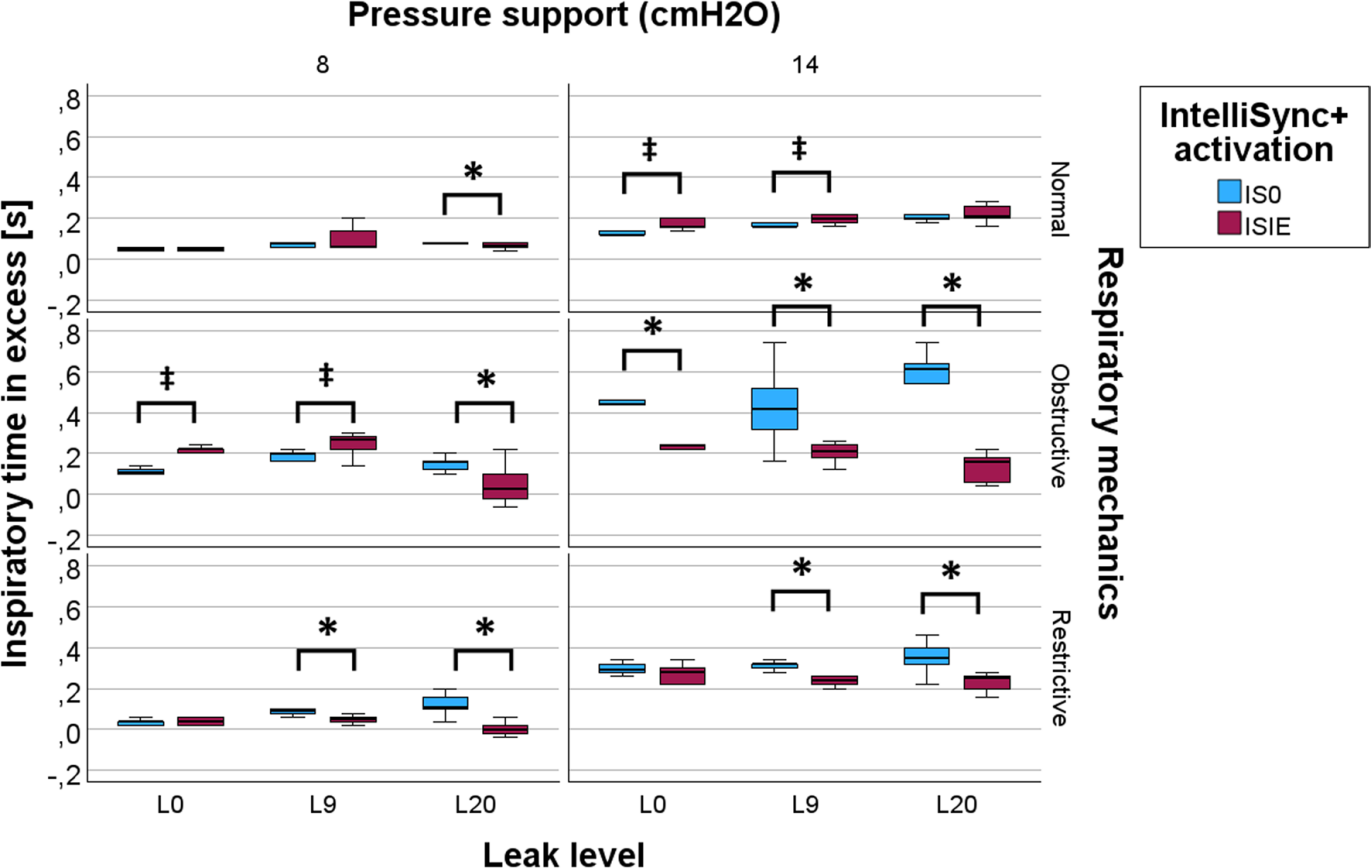
Inspiratory time in excess with and without IntelliSync + ^®^ fully activated in the tested setups. Borders of the colored boxes represent the first and third quartile. The thick line in the middle represents the median. Finally, the lower and upper whisker represent the 5th and 95th percentile, respectively. *p < 0.05, in favor of ISIE. ^‡^p < 0.05 in favor of IS0. *L0* no leak flow, *L9* leak flow of 9 L/min, *L20* leak flow of 20 L/min, *IS0* IntelliSync + ^®^ deactivated, *ISIE* IntelliSync + ^®^ activated during inspiratory and expiratory phase

The Tiex measured with and without IntelliSync + ^®^ fully activated are shown in Fig. 5 for the different experimental conditions. The precise numeric values of Tiex are provided in the *ESM*. In the presence of leaks, ISIE improved Tiex in eight setups but had a detrimental effect in two other setups with a slight increase in Tiex. More in detail, in presence of a small leak (L9), ISIE improved Tiex in obstructive respiratory mechanics with high PS (14 cmH_2_O), and in restrictive respiratory mechanics with both PS levels (8 and 14 cmH_2_O). Conversely, in presence of a small leak, ISIE slightly increased Tiex in normal respiratory mechanics with high PS (14 cmH_2_O) and in obstructive respiratory mechanics with low PS (8 cmH_2_O). In the presence of a large leak (L20), ISIE improved Tiex in normal respiratory mechanics with low PS (8 cmH_2_O) and in obstructive and restrictive respiratory mechanics with both PS levels (8 and 14 cmH_2_O). No increase in Tiex was observed in the presence of a large leak. In the absence of leak (L0), ISIE improved Tiex in obstructive respiratory mechanics with high PS (14 cmH_2_O). A trend towards improved Tiex in restrictive respiratory mechanics with high PS (14 cmH_2_O) was also observed, even if not statistically significant (*p* = 0.247). As with the presence of small leak, in the absence of leak, ISIE slightly increased Tiex in normal respiratory mechanics with high PS (14 cmH_2_O) and in obstructive respiratory mechanics with low PS (8 cmH_2_O).

#### Classical asynchronies

Classical asynchronies observed in this study were auto-triggerings and late cyclings. Illustrations of these asynchronies are shown in the *ESM (eFigure 3).*

##### At steady state

At steady state, classical asynchronies occurred in 6 of the tested setups (17%), always in the presence of leak and mainly (5 from 6 setups) without IntelliSync + ^®^ activation. Classical asynchronies consisted of 97% of auto-triggerings and 2.6% of late cyclings. Five of the six setups with classical asynchronies were with restrictive mechanics. Detailed data is available in *eFigure 4* of the *ESM*. The 6th setup with classical asynchronies at steady state was normal respiratory mechanics, PS 14 cmH_2_O, L20 and IntelliSync + ^®^ not activated. Overall, compared to IS0, ISIE tended to diminish AI even if no statistically significant difference could be demonstrated.

##### Immediately after a leak change

Immediately after a leak increase, during the stabilization period, classical asynchronies occurred in 35 setups (97%), with auto-triggering representing 93.2% and late cycling 6.7% of the classical asynchronies observed. After an important leak increase (from L0 to L20), compared to IS0, ISIE showed a clear trend towards diminishing AI. This result is shown in eFigure 7 in the ESM. When the increase in leak was smaller (L0 to L9 or L9 to L20), the impact of IntelliSync + ^®^ on classical asynchronies was inconstant with either no impact or a slight increase in AI. Detailed results are available in *eTable 9* of the *ESM*. After a leak decrease, classical asynchronies occurred in only 1 (3%) setup: restrictive respiratory mechanics, with PS 8 cmH_2_O, IntelliSync + ^®^ not activated and leak level decrease from L20 to L9 (AI = 30%).

### Comparison of synchrony between partial and full IntelliSync + ^®^ activation (ISI vs ISIE and ISE vs ISIE)

#### Trigger delay

Compared to ISIE, activating IntelliSync + ^®^ only during inspiration (ISI) enabled a slight diminution of Td in restrictive respiratory mechanics with high PS (14 cmH_2_O) and no leak (L0). No other differences on Td were observed between full activation of IntelliSync + ^®^ and its partial activation limited to inspiration. Detailed results are available in *eTables 10–15* in the *ESM*.

#### Inspiratory time in excess

Compared to ISIE, activating IntelliSync +^ ®^ during expiration only (ISE) showed a slight increase in Tiex in normal respiratory mechanics with high PS (14 cmH_2_O) and small leak (L9). No other difference was observed on Tiex between the two activation modalities. Detailed results are available in *eTables 10–15* in the *ESM*.

### Effect of IntelliSync + ^®^ full activation on ventilator pressurization capacity

#### *PTP*_*vent*_* trigger*

In the presence of leak, PTP_vent_trig was improved (decrease in absolute value) by fully activating IntelliSync + ^®^ in 10 conditions (28%). Results are shown in the ESM (*eFigure 5).* PTP_vent_trig was improved in the 5 same setups in presence of small (L9) and large (L20) leaks: normal or obstructive respiratory mechanics with both PS (8 or 14 cmH_2_O) and restrictive respiratory mechanics with high PS (14 cmH_2_O). In the presence of leaks, PTP_vent_trig was never negatively impacted by activating the system. In the absence of leak (L0), ISIE improved PTP_vent_trig in obstructive respiratory mechanics with high PS (14 cmH_2_O). On the other hand, it slightly increased PTP_vent_trig in normal respiratory mechanics with low PS (8 cmH_2_O), and in restrictive respiratory mechanics with low and high PS (8 and 14 cmH_2_O).

#### *PTP*_*vent*_*300*

In the presence of leak, ISIE improved (increased) PTP_vent_300 in 7 setups (19%) and never had a negative effect. Results are shown in the ESM (*eFigure 6).* With small leak (L9) and large leak (L20), PTP_vent_300 was increased by activating IntelliSync + ^®^ in normal respiratory mechanics with both PS levels (8 or 14 cmH_2_O) and in restrictive respiratory mechanics with high PS (14 cmH_2_O). In addition, with small leak (L9), PTP_vent_300 was increased with IntelliSync + ^®^ activated with obstructive respiratory mechanics with low PS (8 cmH_2_O). In the absence of leak (L0), ISIE improved PTP_vent_300 in obstructive respiratory mechanics with high PS (14 cmH_2_O) but altered PTP_vent_300 in normal respiratory mechanics with low PS (8 cmH_2_O).

#### *PTP*_*vent*_*500*

In the presence of leak, ISIE improved PTP_vent_500 in 8 (22%) setups and never had a negative effect. With small (L9) and large leak (L20), PTP_vent_500 was increased by activating IntelliSync + ^®^ in normal respiratory mechanics with both PS levels (8 or 14 cmH_2_O). In addition, in presence of small leak (L9), activation of IntelliSync + ^®^ improved PTP_vent_500 in obstructive respiratory mechanics with low PS (8 cmH_2_O) and restrictive respiratory mechanics with high PS (14 cmH_2_O). With large leak (L20), ISIE also improved PTP_vent_500 in obstructive respiratory mechanics with high PS (14 cmH_2_O). In the absence of leak (L0), ISIE improved PTP_vent_500 in obstructive respiratory mechanics with high PS (14 cmH_2_O) and restrictive respiratory mechanics with low PS (8 cmH_2_O).

## Discussion

In our NIV-PS bench-test, in the presence of leaks, full activation of IntelliSync + ^®^ improved triggering by reducing Td and PTP_vent_trig under different conditions. Expiratory synchrony was also improved, especially with pathological respiratory mechanics with high PS level. Activating IntelliSync + ^®^ also showed a trend towards diminishing classical asynchronies at steady state and, more evidently, after a major leak flow increase. Activating IntelliSync + ^®^ improved pressurization in all measurable conditions. Finally, full IntelliSync + ^®^ activation seemed to combine the advantage of the selective activation on either inspiration or expiration.

Automated systems, when activated in addition to NIV modes, could potentially help optimizing patient–ventilator interaction in the presence of leaks. The Auto-Trak^™^ (Respironics Int., Murrysville PA, USA) that uses real-time curves analysis to automatically optimize expiratory trigger settings allows improving synchrony compared to standard NIV modes in a previous bench study [19]. The IntelliSync + ^®^ system is a more sophisticated automated system that was recently released on the market. Mojoli et al. demonstrated that IntelliSync + ^®^ improved patient–ventilator synchrony in difficult-to-wean invasively ventilated patients [9], a situation where no leaks are present. Our bench study is the first to evaluate the potential benefits of IntelliSync + ^®^, combined with the use of the NIV-PS mode in the presence of leaks.

In clinical practice, NIV is recognized as the first line treatment for acute COPD exacerbation with respiratory acidosis [3]. Applying NIV in this situation is, however, challenging as due to the increased airway resistances, flow decreases slowly during inspiration, leading to frequent late cycling, dynamic air trapping and auto-PEEP. Consequently, as auto-PEEP must first be overcome to trigger the ventilator, prolonged trigger delays and ineffective efforts are favored [20]. Leaks accentuate this phenomenon due to their impact on the flow recorded by the ventilator. This remains true even with NIV modes. Moreover, to increase minute ventilation, high PS level are often needed in COPD patients, further favoring leaks and leak-related asynchronies [7]. In the setup corresponding to acute COPD exacerbation (obstructive respiratory mechanic with high PS), in presence of large leaks, IntelliSync + ^®^ used in addition to the NIV-PS mode was particularly effective in reducing Tiex. Of note, when small leaks were present and low PS level was used with obstructive respiratory mechanics, Tiex increased when IntelliSync + ^®^ was activated. However, using a low level of assistance in acute COPD exacerbation is not standard of care, making this negative impact not clinically relevant. Moreover, the increase in Tiex was limited to 70 ms, which is a short time compared to the duration of expiration, even with tachypnea (expiration duration of about 1300 ms for a respiratory rate of 30/minute). In our bench model, with obstructive respiratory mechanics in the presence of leaks, the impact of IntelliSync + ^®^ on Td was small. The limited effect observed on Td can be attributed to the relatively low inspiratory trigger threshold used with IS0 and with high probability to the absence of dynamic air trapping in our bench model. Based on our results, we think that IntelliSync + ^®^ could reduce Tiex, improve Td and reduce ineffective efforts by limiting the hyperinflation due to poor expiratory synchrony in patients suffering from acute COPD exacerbation. This has, however, to be demonstrated.

Other validated indications for NIV are the treatment of acute pulmonary oedema and prophylactic NIV after various types of surgery [3, 21]. These clinical situations typically involve patients with either normal or restrictive respiratory mechanics, who are ventilated with low levels of pressure support, if not simply continuous positive airway pressure. In setups corresponding to these clinical settings, IntelliSync + ^®^ slightly reduced Td in normal respiratory mechanics. It also improved Tiex in both normal and restrictive mechanics with low PS levels, in the presence of both small and large leaks. In practice, prolonged Td and non-optimal Tiex are not major concerns in these clinical situations, and the advantage of activating IntelliSync + ^®^ may here have a questionable clinical impact. However, importantly, activating IntelliSync + ^®^ never had a negative effect on either Td or Tiex, indicating that the system could be used safely when treating pulmonary edema or delivering prophylactic NIV. In clinical practice, one frequent problem in these situations is the occurrence of auto-triggerings, which can cause discomfort and intolerance to treatment. In our bench test, auto-triggerings were observed at steady state, especially with restrictive respiratory mechanics when IntelliSync + ^®^ was not activated. The high incidence of auto-triggerings in these situations may partially be explained by a relatively low inspiratory trigger threshold during IS0 (1.5 L/min) that may have contributed to destabilizing the system. It must, however, be noted that this is a commonly used inspiratory trigger threshold in clinical practice. Activating IntelliSync + ^®^ reduced auto-triggering in nearly all the setups. In cases of sudden major increase in leak amplitude, which is common during NIV, auto-triggerings were very frequent in the absence of IntelliSync + ^®^ and markedly reduced with it. Our results on auto-triggerings tend to suggest that the correct detection of the inspiratory effort was better with the waveform method used by IntelliSync + ^®^, compared to the usual approach of setting a flow-trigger threshold.

The last situation where NIV might be used is early during acute hypoxemic respiratory failure, even if this indication is more debated [22]. Patients could have normal or restrictive respiratory mechanics and receive either low or high PS levels, depending on the clinical situation. The only specific setup that was not previously discussed is delivering high PS levels in the presence of normal or restrictive mechanics. In our bench model, activating IntelliSync + ^®^ in this situation reduced Td and improved Tiex in presence of large leaks in restrictive respiratory mechanics. For Tiex, with small leaks, the beneficial effect of activating IntelliSync + ^®^ was also present with restrictive mechanics. In the same situation, with normal mechanics, IntelliSync + ^®^ slightly altered Tiex but not enough to have any significant clinical impact. Overall, when high levels of PS are used in the presence of restrictive or normal respiratory mechanics, activating IntelliSync + ^®^ may also help improve patient–ventilator synchrony in patients treated with NIV.

Regardless of the reason for using NIV, good pressurization is important to allow better matching between the patient’s demand and the ventilator inspiratory flow, as this improves comfort and reduces the risk of NIV failure [23]. Overall, in presence of leaks, activating IntelliSync + ^®^ was associated with improved pressurization both during the triggering phase and the pressurization phase. The PTP_vent_trig improvement is probably due to the ability of IntelliSync + ^®^ to detect earlier the start of the inspiratory effort (Td is overall reduced when IntelliSync + ^®^ is activated) compared to the usual pneumatic trigger. We can hypothesize that the waveform method used by IntelliSync + ^®^, which uses a change in form of the ventilatory curve, is more sensitive to detect an inspiratory effort compared to the detection of a change in flow or pressure amplitude in the ventilator circuit. This could contribute to improving the pressurization in the early breath phase. As the calculation of PTP_vent_300 and PTP_vent_500 includes the subtraction of the absolute value of PTP_vent_trig, diminished absolute values of PTP_vent_trig probably explain, at least partially, the improved pressurization indexes [17].

In clinical practice, IntelliSync + ^®^ can be fully (inspiration and expiration) or partially (inspiration or expiration) activated by selecting the corresponding option on the ventilator screen. In our bench test, full activation of IntelliSync + ^®^ seemed to combine the advantage of partial activation on inspiration and expiration. From a clinical point of view, our results thus suggest that full activation could be used as the default mode when using IntelliSync + ^®^.

All in all, this bench study enabled to attest the potential benefits on patient–ventilator synchrony of IntelliSync + ^®^ in the presence of leaks, which was a desirable step before testing it on patients in clinical practice. Importantly, we could also identify respiratory mechanics which most likely could benefit from IntelliSync + ^®^.

### Study limitations

This study has some limitations. First, as a bench study, it cannot be directly inferred to clinical situations. Indeed, the impact of IntelliSync + ^®^ may be less predictable if the leak level and respiratory pattern show more variability, like in real clinical setting. Importantly, we did not use a PVC head equipped with a face mask to simulate the NIV interface but created calibrated leaks in tested ventilator circuit, as close as possible to the connection of the lung model. The reason for that, was to compare similar calibrated leaks levels between the different tested conditions. However, this approach does not allow taking into account the fact that leaks at the NIV interface can vary sometimes unpredictably during inspiration and is thus clearly a limitation of our model. Second, our bench model was designed to avoid dynamic air trapping, which can lead to increased Td. It is thus possible that our model underestimated the effect of IntelliSync + ^®^ on the triggering phase in some situations. However, even in the absence of air trapping, we demonstrated the ability of IntelliSync + ^®^ to reduce Td in the presence of leaks. Third, the inspiratory trigger set at 1.5 L/min was relatively low, even if consistent with the literature [14] and clinical use. This could have increased the incidence of auto-triggering in the presence of leaks. Fourth, as only a valve Hamilton ventilator was used during this study (Hamilton S1-Ventilator), our results are not generalizable for other ventilator types, like turbine ventilators. Finally, the sampling frequency of 50 Hz used for the recordings could not detect differences smaller than 20 ms. However, differences in Td or Tiex of less than 20 ms are not clinically relevant.

## Conclusions

In this bench study, we demonstrated a potential benefit of using IntelliSync + ^®^, in addition to the NIV-PS mode, to improve synchrony inspiratory and expiratory patient–ventilator synchrony in the presence of leaks. The system was particularly effective in case of obstructive respiratory mechanic with high PS level, which corresponds to acute COPD exacerbation and in case of restrictive mechanics and high-pressure support level, which corresponds to acute hypoxemic respiratory failure. Activating IntelliSync + ^®^ also tended to decrease classical asynchronies and improved ventilator pressurization. Overall, this bench study provides preclinical data suggesting a potential favorable impact of IntelliSync + ^®^ in the presence of leaks. This allows now designing a clinical trial to assess the impact of the system in patients undergoing NIV.

## Supplementary Information

Supplementary material 1

## Data Availability

The data sets used and/or analyzed during the current study are available from the corresponding author on reasonable request.

## Abbreviations

AI: Asynchrony index
APE: Acute pulmonary edema
APRV: Airway pressure release ventilation mode
COPD: Chronic obstructive pulmonary disease
DV: Driver ventilator
ESM: Electronic supplementary material
IS0: IntelliSync + ^®^ deactivated
ISE: IntelliSync + ^®^ activated during expiration
ISI: IntelliSync + ^®^ activated during inspiration
ISIE: IntelliSync + ^®^ activated during inspiration and expiration
L0: Leak flow of 0 L/min
L9: Leak flow of 9 L/min
L20: Leak flow of 20 L/min
NIV: Non-invasive ventilation
PEEP: Positive end expiratory pressure
PS: Pressure support
Pt: Pressurization time
PTP_vent_300: Pressure–time product of the ventilator at 300 ms
PTP_vent_500: Pressure–time product of the ventilator at 500 ms
PTP_vent_trig: Triggering pressure–time product of the ventilator
SV: Second ventilator
Td: Trigger delay
Tiex: Inspiratory time in excess

## References

[1] Vignaux L, Tassaux D, Jolliet P (2007) Performance of noninvasive ventilation modes on ICU ventilators during pressure support: a bench model study. Intensive Care Med 33(8):1444–1451 17563875 10.1007/s00134-007-0713-0

[2] Antonelli M, Conti G, Moro ML, Esquinas A, Gonzalez-Diaz G, Confalonieri M et al (2001) Predictors of failure of noninvasive positive pressure ventilation in patients with acute hypoxemic respiratory failure: a multi-center study. Intensive Care Med 27(11):1718–1728 11810114 10.1007/s00134-001-1114-4

[3] Rochwerg B, Brochard L, Elliott MW, Hess D, Hill NS, Nava S et al (2017) Official ERS/ATS clinical practice guidelines: noninvasive ventilation for acute respiratory failure. Eur Respir J 50(2):1602426 28860265 10.1183/13993003.02426-2016

[4] Carlucci A, Richard JC, Wysocki M, Lepage E, Brochard L, SRLF Collaborative Group on Mechanical Ventilation (2001) Noninvasive versus conventional mechanical ventilation: an epidemiologic survey. Am J Respir Crit Care Med 163(4):874–880 11282759 10.1164/ajrccm.163.4.2006027

[5] Brochard L, Mancebo J, Wysocki M, Lofaso F, Conti G, Rauss A et al (1995) Noninvasive ventilation for acute exacerbations of chronic obstructive pulmonary disease. N Engl J Med 333(13):817–822 7651472 10.1056/NEJM199509283331301

[6] Vignaux L, Vargas F, Roeseler J, Tassaux D, Thille AW, Kossowsky MP et al (2009) Patient–ventilator asynchrony during non-invasive ventilation for acute respiratory failure: a multicenter study. Intensive Care Med 35(5):840 19183949 10.1007/s00134-009-1416-5

[7] Vignaux L, Tassaux D, Carteaux G, Roeseler J, Piquilloud L, Brochard L et al (2010) Performance of noninvasive ventilation algorithms on ICU ventilators during pressure support: a clinical study. Intensive Care Med 36(12):2053–2059 20689921 10.1007/s00134-010-1994-2

[8] Mojoli F, Pozzi M, Orlando A, Bianchi IM, Arisi E, Iotti GA et al (2022) Timing of inspiratory muscle activity detected from airway pressure and flow during pressure support ventilation: the waveform method. Crit Care 30(26):32 10.1186/s13054-022-03895-4 PMC8802480 35094707

[9] Mojoli F, Orlando A, Bianchi IM, Puce R, Arisi E, Salve G et al (2022) Waveforms-guided cycling-off during pressure support ventilation improves both inspiratory and expiratory patient-ventilator synchronisation. Anaesth Crit Care Pain Med 41(6):101153 36084912 10.1016/j.accpm.2022.101153

[10] Thille AW, Lyazidi A, Richard JCM, Galia F, Brochard L (2009) A bench study of intensive-care-unit ventilators: new versus old and turbine-based versus compressed gas-based ventilators. Intensive Care Med 35(8):1368–1376 19352622 10.1007/s00134-009-1467-7 PMC2873304

[11] Delgado C, Romero JE, Puig J, Izquierdo A, Ferrando C, Belda FJ et al (2017) Performance of the new turbine mid-level critical care ventilators. Respir Care 62(1):34–41 28003552 10.4187/respcare.04938

[12] Sheehy RD, Duce B, Edwards TP, Churton JA, Sharma R, Hukins CA (2020) Double-triggering during noninvasive ventilation in a simulated lung model. Respir Care 65(9):1333–1338 32184378 10.4187/respcare.07280

[13] Moerer O, Harnisch LO, Herrmann P, Zippel C, Quintel M (2016) Patient-ventilator interaction during noninvasive ventilation in simulated COPD. Respir Care 61(1):15–22 26556898 10.4187/respcare.04141

[14] Carteaux G, Lyazidi A, Cordoba-Izquierdo A, Vignaux L, Jolliet P, Thille AW et al (2012) Patient-ventilator asynchrony during noninvasive ventilation. Chest 142(2):367–376 22406958 10.1378/chest.11-2279

[15] Vignaux L, Piquilloud L, Tourneux P, Jolliet P, Rimensberger PC (2014) Neonatal and adult ICU ventilators to provide ventilation in neonates, infants, and children: a bench model study. Respir Care 59(10):1463–1475 25118306 10.4187/respcare.02540

[16] Piquilloud L, Tassaux D, Bialais E, Lambermont B, Sottiaux T, Roeseler J et al (2012) Neurally adjusted ventilatory assist (NAVA) improves patient–ventilator interaction during non-invasive ventilation delivered by face mask. Intensive Care Med 38(10):1624–1631 22885649 10.1007/s00134-012-2626-9

[17] Olivieri C, Costa R, Conti G, Navalesi P (2012) Bench studies evaluating devices for non-invasive ventilation: critical analysis and future perspectives. Intensive Care Med 38(1):160–167 22124770 10.1007/s00134-011-2416-9

[18] Thille AW, Rodriguez P, Cabello B, Lellouche F, Brochard L (2006) Patient-ventilator asynchrony during assisted mechanical ventilation. Intensive Care Med 32(10):1515–1522 16896854 10.1007/s00134-006-0301-8

[19] Chen Y, Cheng K, Zhou X (2016) Effectiveness of inspiratory termination synchrony with automatic cycling during noninvasive pressure support ventilation. Med Sci Monit 20(22):1694–1701 10.12659/MSM.896059 PMC4915317 27198165

[20] Tassaux D, Gainnier M, Battisti A, Jolliet P (2005) Impact of expiratory trigger setting on delayed cycling and inspiratory muscle workload. Am J Respir Crit Care Med 172(10):1283–1289 16109983 10.1164/rccm.200407-880OC

[21] Jaber S, Chanques G, Jung B, Riou B (2010) Postoperative noninvasive ventilation. Anesthesiology 112(2):453–461 20068454 10.1097/ALN.0b013e3181c5e5f2

[22] Grasselli G, Calfee CS, Camporota L, Poole D, Amato MBP, Antonelli M et al (2023) ESICM guidelines on acute respiratory distress syndrome: definition, phenotyping and respiratory support strategies. Intensive Care Med 49(7):727–759 37326646 10.1007/s00134-023-07050-7 PMC10354163

[23] Chiumello D, Pelosi P, Croci M, Bigatello LM, Gattinoni L (2001) The effects of pressurization rate on breathing pattern, work of breathing, gas exchange and patient comfort in pressure support ventilation. Eur Respir J 18(1):107–114 11510780 10.1183/09031936.01.00083901

