# Supplementary material for: Patient–ventilator synchrony under non-invasive ventilation is improved by an automated real time waveform analysis algorithm: a bench study
Source: Intensive Care Med Exp. 2025 Feb 12;13:16. doi: 10.1186/s40635-025-00726-y (PMC11822138; doi:10.1186/s40635-025-00726-y)
Supplement: Supplementary file 1 — Supplementary material 1 [file 40635_2025_726_MOESM1_ESM.docx]

**Patient-Ventilator Synchrony Under Non-Invasive Ventilation Is Improved Using an Automated Real Time Waveform Guided Algorithm (IntelliSync+®) – A Bench Study**

*Electronic Supplementary Material (ESM)*

Table of content

[1. Methodology 2](#_Toc184055525)

[*1.1.* *Leak generation, detailed description of the system used* 2](#_Toc184055526)

[*1.1.1.* *Continuous leak* 2](#_Toc184055527)

[*1.1.2.* *Inspiratory only leak* 2](#_Toc184055528)

[*1.2.* *Effective leaks measured* 3](#_Toc184055529)

[*1.3.* *Driver ventilator settings* 4](#_Toc184055530)

[*1.4.* *Compliance and resistance settings* 5](#_Toc184055531)

[*1.5.* *Details on the measurement of PTP_vent_trig, PTP_vent_300 and PTP_vent_500* 5](#_Toc184055532)

[*1.6.* *Classical asynchronies observed* 6](#_Toc184055533)

[2. Detailed results 8](#_Toc184055534)

[*2.1.* *IS0 vs ISIE at steady state* 8](#_Toc184055535)

[*2.2.* *Major asynchronies IS0 vs ISIE Post-modification* 15](#_Toc184055536)

[*2.3.* *ISI vs ISIE and ISE vs ISIE at steady state* 15](#_Toc184055537)

[3. Bibliography 22](#_Toc184055538)

1. Methodology
   1. *Leak generation, detailed description of the system used*
      1. *Continuous leak*

Continuous leaks were generated to evaluate triggering asynchronies. A 22-22 Male-Female connector with a 7.6mm port (Intersurgical, East Syracuse, NY, USA) was inserted between the Y-piece and the second compartment of the test lung. The port was connected to an Optimus suction connecting tube Ø 4mm (Promedical AG, Glarus, Switzerland), the length of which enabled to modulate leak level. If the needed length was too important to generate the targeted small leak and thus became impractical, a suction tubing connector was added at the end of the tubing to increase resistance to airflow. The two tubings calibrated to respectively generate small and large leaks were organized in Y. A clamp forceps was then used to open only one circuit at a time. **eFigure 1** illustrates the continuous leak system.


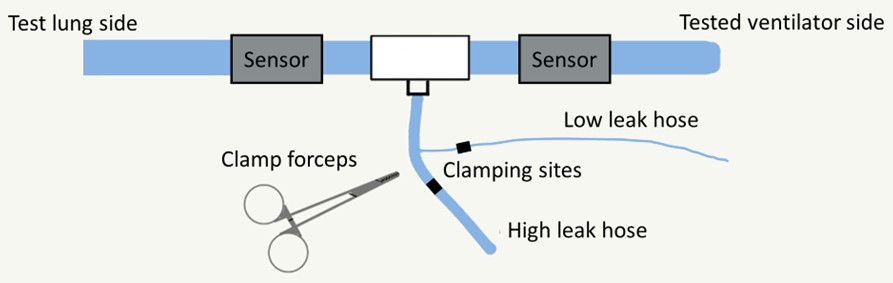


**eFigure 1**: Continuous leak system.

- - 1. *Inspiratory only leak*

Inspiratory leaks were generated in order to evaluate cycling off asynchronies. A 22-22 Male-Female connector with a 7.6mm port was placed between the Y-piece and the test lung. A NIV circuit immersed in a water column contained in an hermetically sealed recipient was connected to it. The variation of water column height, as well as the modulation of the flow through the hose enabling air exit allowed to generate two different leak flows. A plastic connector enabled to create two air circuits, one for each leak flow. By using a clamp forceps on the hose enabling air exit, we could open only one circuit at the time. **eFigure 2** illustrates the inspiratory leak system.


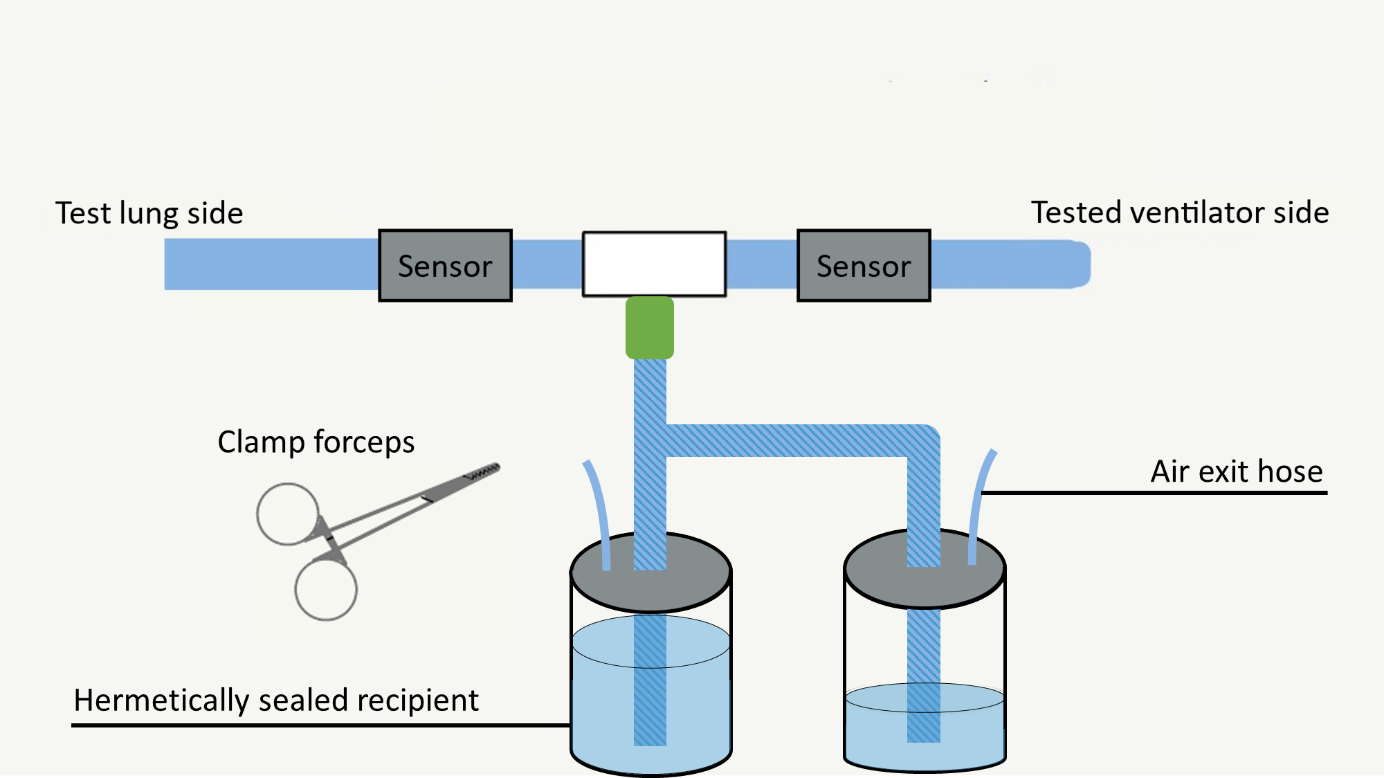


**eFigure 2**: Inspiratory only leak system.

- 1. *Effective leaks measured*

Two flow and pressure sensors were placed before and after the leak, to measure the effective leak flows obtained for low leak and high leak.

For continuous leaks, the targeted flow was reached by adapting the length of the hose responsible for the leak. Effective mean inspiro-expiratory leaks flow were calculated breath by breath, as the difference between inspiratory and expiratory volumes (measured by integrating the flow-time signal recorded by sensor (a), Error! Reference source not found. of the article), divided by the total duration of the ventilatory cycle. Ten ventilatory cycles were studied in NIV mode with total deactivation of IntelliSync+®, a mean leak flow of 9L/min (L9), respectively 20L/min (L20), were aimed. After each leak calibration, the absence of leak when the leak is closed (L0) was attested. **eTable 1** shows the actual continuous leak values obtained during data collection.

| **Respiratory**  **mechanics** | **PS [cmH_2_O]** | **Leak level** | | |
| --- | --- | --- | --- | --- |
|  |  | **L0 [L/min]** | **L9 [L/min]** | **L20 [L/min]** |
| **Normal** | **8** | -0.20 [-0.29 - -0.11] | 9.72 [9.64 - 10.07] | 20.10 [19.81 - 20.42] |
|  | **14** | -0.44 [-0.49 - -0.38] | 10.67 [10.32 - 10.99] | 22.53 [21.97 - 22.80] |
| **Obstructive** | **8** | -0.45 [-0.50 - -0.37] | 9.25 [9.05 - 9.66] | 21.60 [21.35 - 22.10] |
|  | **14** | -0.43 [-0.49 - -0.34] | 8.74 [8.46 - 8.86] | 20.86 [20.32 - 21.32] |
| **Restrictive** | **8** | -0.26 [-0.38 - -0.16] | 9.64 [9.23 - 9.87] | 22.77 [22.26 - 23.14] |
|  | **14** | -0.34 [-0.37 - -0.28] | 10.25 [10.00 - 10.51] | 21.88 [21.55 - 22.15] |

**eTable 1:** Actual continuous leak level obtained during data collection, according to ventilatory mechanics and pressure support. At L0, aim was a 0L/min leak. At L9, aim was a 9L/min leak. At L20, aim was a 20L/min leak. PS: Pressure support.

For inspiratory leak, the targeted flow were reached by adapting water height and, if necessary, the length of the hose enabling air outlet of the sealed recipient. Effective leaks limited to inspiration for each tested set-up were computed in L/min as the difference in inspiratory volumes measured before (sensor (a), Error! Reference source not found. of the article) and after (sensor (b), Error! Reference source not found. of the article) the leak, divided by the pressurization time. Ten ventilatory cycles were studied in NIV mode with total deactivation of IntelliSync+®, a mean leak during the inspiration phase of 9L/min (L9), respectively 20L/min (L20), were aimed. After each leak calibration, the absence of leak when the leak was closed (L0) was attested. This process was repeated for all pressure supports and ventilatory mechanics. **eTable 2** shows the actual inspiratory leak levels obtained during data collection.

| **Respiratory mechanics** | **PS [cmH_2_O]** | **Leak level** | | |
| --- | --- | --- | --- | --- |
|  |  | **L0 [L/min]** | **L9 [L/min]** | **L20 [L/min]** |
| **Normal** | **8** | 1,19 [0.99 - 1.34] | 9,98 [9.42 - 10.47] | 21,56 [20.65 - 22.44] |
|  | **14** | 0,62 [0.42 - 0.82] | 9,17 [8.75 - 9.64] | 22,05 [21.05 - 22.89] |
| **Obstructive** | **8** | 1,59 [1.41 - 1.82] | 9,87 [9.51 - 10.35] | 22,42 [20.70 - 23.46] |
|  | **14** | 1,10 [0.97 - 1.27] | 10,49 [9.79 - 11.21] | 20,30 [19.05 - 21.73] |
| **Restrictive** | **8** | 2,01 [1.88 - 2.19] | 9,60 [9.29 - 9.85] | 19,64 [18.13 - 20.45] |
|  | **14** | 1,62 [1.21 - 1.81] | 9,44 [9.15 - 9.77] | 20,09 [19.37 - 21.09] |

**eTable 2:** Actual inspiratory leak level obtained during data collection, according to ventilatory mechanics and pressure support. At L0, aim was a 0L/min leak. At L9, aim was a 9L/min leak. At L20, aim was a 20L/min leak. PS: Pressure support

- 1. *Driver ventilator settings*

The driver ventilator was set in a pressure regulated mode to mimic physiological respiration. In practice we used airway pressure release ventilation (APRV) mode as it was the most convenient to parameter the breaths.

High pressure time and low pressure time were set in order to enable a moderately increased respiratory rate without generating auto-PEEP and while keeping physiological I:E ratio.

High pressure and pressurization slope were set in order to reach predetermined moderately increased occlusion pressure at 100 ms (P0.1) and global occlusion pressure (Poccl). Low pressure was set at the highest level before the metal strip touched the edge of the second compartment. This enabled a direct reactivity: when the first compartment was inflated, the metal strip very early dragged the edge of the second compartment up. The value did not equate the value of PEEP set on the second ventilator because of the difference in compliance between the two compartments of the test lung.

- 1. *Compliance and resistance settings*

Compliance was set directly on the second compartment of the test lung, using the in-built spring system, and was checked by measuring static compliance with the second ventilator set in volume control mode. Compliance values used for restrictive respiratory mechanics were in line with previous published bench tests, and aimed to simulate a moderate compliance reduction, as usually observed in patients treated with NIV [1,2]. Resistance was increased to simulate obstructive respiratory mechanics by inserting a PneuFlo^®^ Parabolic Resistor Rp20 (Michigan Instruments, Grand Rapids, USA) in the second ventilator circuit, as close as possible to the second compartment of the Michigan test lung. Mean total resistance was measured for each respiratory mechanics with the second ventilator set in volume control mode with a constant inspiratory flow of 60 L/min (or 1L/s) in the absence of leak. Resistance value of obstructive respiratory mechanics was in line with previous published works, and aimed to simulate a moderate resistance increase [1,3–5].

- 1. *Details on the measurement of PTP_vent_trig, PTP_vent_300 and PTP_vent_500*

Ventilatory pressure time product of the triggering phase (PTP_vent_trig, A in **Figure 2** of the article), corresponds to the area between the pressure-time curve recorded in the second ventilator circuit and a virtual line corresponding to the baseline airway pressure (end-expiratory pressure), from the start of the inspiratory effort (initial pressure drop) to the point of return to baseline airway pressure [6]. Ventilatory pressure time product at 300 ms and 500 ms (PTP_vent_300 and PTP_vent_500), that are indices of the pressurization capacity of the second ventilator, are calculated as follows: computation of the area between the pressure-time curve and the baseline airway pressure virtual line, from the point of return to baseline airway pressure (i.e. end of the PTP_vent_trig measurement area) to 300 ms of pressurization, respectively 500 ms, minus the absolute value of PTP_vent_trig (B and C in **Figure 2** of the article) [6].

- 1. *Classical asynchronies observed*

Hereafter, **eFigure 3**, illustrates the curves of the observed classical asynchronies in this study.


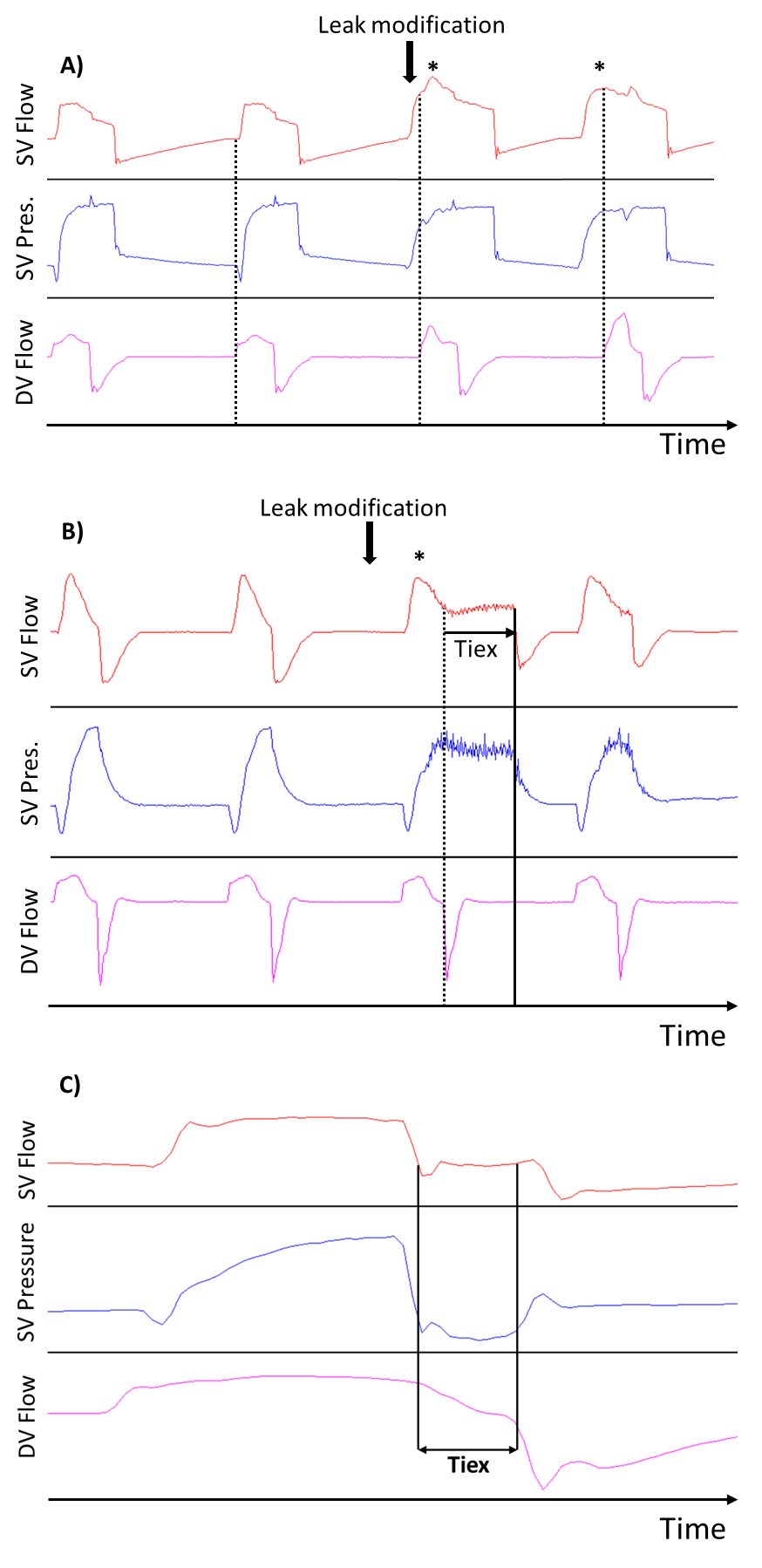


**eFigure 3**: Illustration of the asynchronies observed during this bench test study.
A) Auto-triggerings in the third and fourth breathing cycles. The vertical dashed lines illustrate the start of neural inspiration. B) Late cycling in the third respiratory cycle. The vertical dashed line represents end of neural inspiration, the vertical full line represents end of pressurization by the second ventilator. Artefacts visible in the 3rd and 4th cycles of the second ventilator are due to bubbles created in the water column by the leaks limited to inspiration, after change in the leak flow from L0 to L20. C) An example of negative inspiratory time in excess which does not meet the predefined criterion for classical asynchrony. *Classical asynchronous cycle. Tiex: inspiratory time in excess; SV: second ventilator; DV: driver ventilator.

1. Detailed results
   1. *IS0 vs ISIE at steady state*

**eTable 3** to **eTable 8** present the results obtained during our study, at steady state.

| **Normal respiratory mechanics, 8 cmH2O** | | | | |
| --- | --- | --- | --- | --- |
| **L0** | **IS0** | **ISIE** | **P value** |  |
| **Trigger delay [ms]** | 120 [100 - 120] | 120 [120 - 120] | 0.481 |  |
| **Inspiratory time in excess [s]** | 0.05 [0.04 – 0.06] | 0.05 [0.04 – 0.06] | 1.000 |  |
| **PTP_vent_ trigger [cmH_2_O*s]** | 0.538 [0.522 - 0.544] | 0.575 [0.561 - 0.588] | 0.004 |  |
| **PTP_vent_ 300 [cmH_2_O*s]** | -0.472 [-0.494 - -0.454] | -0.513 [-0.531 - -0.503] | 0.009 |  |
| **PTP_vent_ 500 [cmH_2_O*s]** | 0.149 [0.102 - 0.198] | 0.132 [0.074 - 0.146] | 0.218 |  |
| **Classical asynchronies** |  | | | |
| - **Auto-triggering [%]** | 0 | 0 | 1.000 |  |
| - **Early cycling [%]** | 0 | 0 | 1.000 |  |
| - **Delayed cycling [%]** | 0 | 0 | 1.000 |  |
| **L9** | **IS0** | **ISIE** | **P value** |  |
| **Trigger delay [ms]** | 100 [100 - 120] | 120 [100 - 120] | 0.481 |  |
| **Inspiratory time in excess [s]** | 0.08 [0.07 – 0.08] | 0.06 [0.06 – 0.12] | 0.481 |  |
| **PTP_vent_ trigger [cmH_2_O*s]** | 0.503 [0.473 - 0.510] | 0.403 [0.397 - 0.411] | <0.001 |  |
| **PTP_vent_ 300 [cmH_2_O*s]** | -0.468 [-0.481 - -0.450] | -0.384 [-0.385 - -0.375] | <0.001 |  |
| **PTP_vent_ 500 [cmH_2_O*s]** | -0.009 [-0.046 - 0.048] | 0.076 [0.014 - 0.100] | 0.019 |  |
| **Classical asynchronies** |  | | | |
| - **Auto-triggering [%]** | 0 | 0 | 1.000 |  |
| - **Early cycling [%]** | 0 | 0 | 1.000 |  |
| - **Delayed cycling [%]** | 0 | 0 | 1.000 |  |
| **L20** | **IS0** | **ISIE** | **P value** |  |
| **Trigger delay [ms]** | 110 [100 - 120] | 60 [60 - 60] | <0.001 |  |
| **Inspiratory time in excess [s]** | 0.08 [0.08 – 0.08] | 0.07 [0.06 – 0.08] | 0.023 |  |
| **PTP_vent_ trigger [cmH_2_O*s]** | 0.436 [0.411 - 0.441] | 0.278 [0.272 - 0.285] | <0.001 |  |
| **PTP_vent_300 [cmH_2_O*s]** | -0.429 [-0.437 - -0.410] | -0.267 [-0.272 - -0.259] | <0.001 |  |
| **PTP_vent_ 500 [cmH_2_O*s]** | -0.081 [-0.111 - -0.056] | 0.089 [0.047 - 0.101] | <0.001 |  |
| **Classical asynchronies** |  | | | |
| - **Auto-triggering [%]** | 0 | 0 | 1.000 |  |
| - **Early cycling [%]** | 0 | 0 | 1.000 |  |
| - **Delayed cycling [%]** | 0 | 0 | 1.000 |  |

**eTable 3:** Results at steady state for a normal ventilatory mechanics and an pressure support of 8cmH_2_O. L0 : no leak; L9 : leak flow of 9L/min; L20 : leak flow of 20L/min. IS0 : IntelliSync+® deactivated; ISIE : IntelliSync+® activated during inspiratory and expiratory phase.

| **Normal respiratory mechanics, 14 cmH2O** | | | |
| --- | --- | --- | --- |
| **L0** | **IS0** | **ISIE** | **P value** |
| **Trigger delay [ms]** | 100 [100 - 100] | 100 [100 - 100] | 1.000 |
| **Inspiratory time in excess [s]** | 0.12 [0.12 – 0.14] | 0.16 [0.16 – 0.20] | <0.001 |
| **PTP_vent_ trigger [cmH_2_O*s]** | 0.363 [0.342 - 0.384] | 0.396 [0.365 - 0.399] | 0.105 |
| **PTP_vent_300 [cmH_2_O*s]** | -0.005 [-0.044 - 0.045] | -0.059 [-0.093 - 0.004] | 0.247 |
| **PTP_vent_ 500 [cmH_2_O*s]** | 1.519 [1.443 - 1.638] | 1.485 [1.432 - 1.583] | 0.529 |
| **Classical asynchronies** |  | | |
| - **Auto-triggering [%]** | 0 | 0 | 1.000 |
| - **Early cycling [%]** | 0 | 0 | 1.000 |
| - **Delayed cycling [%]** | 0 | 0 | 1.000 |
| **L9** | **IS0** | **ISIE** | **P value** |
| **Trigger delay [ms]** | 100 [100 - 120] | 100 [100 - 120] | 1.000 |
| **Inspiratory time in excess [s]** | 0.16 [0.16 – 0.18] | 0.20 [0.19 – 0.22] | 0.001 |
| **PTP_vent_ trigger [cmH_2_O*s]** | 0.358 [0.343 - 0.364] | 0.273 [0.257 - 0.282] | <0.001 |
| **PTP_vent_300 [cmH_2_O*s]** | -0.123 [-0.161 - -0.080] | 0.010 [-0.033 - 0.043] | <0.001 |
| **PTP_vent_ 500 [cmH_2_O*s]** | 1.083 [1.025 - 1.217] | 1.319 [1.270 - 1.375] | 0.002 |
| **Classical asynchronies** |  | | |
| - **Auto-triggering [%]** | 0 | 0 | 1.000 |
| - **Early cycling [%]** | 0 | 0 | 1.000 |
| - **Delayed cycling [%]** | 0 | 0 | 1.000 |
| **L20** | **IS0** | **ISIE** | **P value** |
| **Trigger delay [ms]** | 100 [80 - 100] | 60 [60 - 60] | <0.001 |
| **Inspiratory time in excess [s]** | 0.20 [0.20 – 0.22] | 0.21 [0.20 – 0.26] | 0.315 |
| **PTP_vent_ trigger [cmH_2_O*s]** | 0.310 [0.301 - 0.318] | 0.175 [0.167 - 0.180] | <0.001 |
| **PTP_vent_300 [cmH_2_O*s]** | -0.106 [-0.134 - -0.079] | 0.073 [0.049 - 0.130] | <0.001 |
| **PTP_vent_ 500 [cmH_2_O*s]** | 1.024 [0.945 - 1.081] | 1.232 [1.191 - 1.339] | <0.001 |
| **Classical asynchronies** |  | | |
| - **Auto-triggering [%]** | 0 | 0 | 1.000 |
| - **Early cycling [%]** | 0 | 0 | 1.000 |
| - **Delayed cycling [%]** | 0 | 0 | 1.000 |

**eTable 4**: Results at steady state for normal ventilatory mechanics and an pressure support of 14cmH_2_O. L0 : no leak; L9 : leak flow of 9L/min; L20 : leak flow of 20L/min. IS0 : IntelliSync+® deactivated; ISIE : IntelliSync+® activated during inspiratory and expiratory phase.

| **Obstructive respiratory mechanics, 8 cmH2O** | | | |
| --- | --- | --- | --- |
| **L0** | **IS0** | **ISIE** | **P value** |
| **Trigger delay [ms]** | 80 [80 - 100] | 100 [90 - 100] | 0.143 |
| **Inspiratory time in excess [s]** | 0.11 [0.10 – 0.12] | 0.22 [0.21 – 0.22] | <0.001 |
| **PTP_vent_ trigger [cmH_2_O*s]** | 0.293 [0.283 - 0.306] | 0.271 [0.248 - 0.298] | 0.190 |
| **PTP_vent_300 [cmH_2_O*s]** | 0.233 [0.187 - 0.244] | 0.238 [0.186 - 0.272] | 0.684 |
| **PTP_vent_ 500 [cmH_2_O*s]** | 1.436 [1.387 - 1.474] | 1.403 [1.399 - 1.444] | 0.579 |
| **Classical asynchronies** |  | | |
| - **Auto-triggering [%]** | 0 | 0 | 1.000 |
| - **Early cycling [%]** | 0 | 0 | 1.000 |
| - **Delayed cycling [%]** | 0 | 0 | 1.000 |
| **L9** | **IS0** | **ISIE** | **P value** |
| **Trigger delay [ms]** | 80 [80 - 80] | 60 [60 - 80] | 0.004 |
| **Inspiratory time in excess [s]** | 0.20 [0.17 – 0.20] | 0.27 [0.23 – 0.28] | 0.003 |
| **PTP_vent_ trigger [cmH_2_O*s]** | 0.208 [0.194 - 0.235] | 0.153 [0.139 - 0.162] | <0.001 |
| **PTP_vent_300 [cmH_2_O*s]** | 0.161 [0.131 - 0.228] | 0.272 [0.213 - 0.272] | 0.023 |
| **PTP_vent_ 500 [cmH_2_O*s]** | 1.193 [1.153 - 1.318] | 1.358 [1.281 - 1.376] | 0.023 |
| **Classical asynchronies** |  | | |
| - **Auto-triggering [%]** | 0 | 0 | 1.000 |
| - **Early cycling [%]** | 0 | 0 | 1.000 |
| - **Delayed cycling [%]** | 0 | 0 | 1.000 |
| **L20** | **IS0** | **ISIE** | **P value** |
| **Trigger delay [ms]** | 60 [60 - 80] | 60 [60 - 80] | 0.739 |
| **Inspiratory time in excess [s]** | 0.16 [0.13 – 0.16] | 0.03 [-0.01 – 0.09] | 0.003 |
| **PTP_vent_ trigger [cmH_2_O*s]** | 0.136 [0.128 - 0.151] | 0.102 [0.088 - 0.106] | <0.001 |
| **PTP_vent_300 [cmH_2_O*s]** | 0.232 [0.226 - 0.271] | 0.257 [0.225 - 0.285] | 0.739 |
| **PTP_vent_ 500 [cmH_2_O*s]** | 1.255 [1.163 - 1.311] | 1.223 [1.140 - 1.272] | 0.631 |
| **Classical asynchronies** |  | | |
| - **Auto-triggering [%]** | 5 | 0 | 0.667 |
| - **Early cycling [%]** | 0 | 0 | 1.000 |
| - **Delayed cycling [%]** | 0 | 0 | 1.000 |

**eTable 5**: Results at steady state for obstructive ventilatory mechanics and an pressure support of 8cmH_2_O. L0 : no leak; L9 : leak flow of 9L/min; L20 : leak flow of 20L/min. IS0 : IntelliSync+® deactivated; ISIE : IntelliSync+® activated during inspiratory and expiratory phase.

| **Obstructive respiratory mechanics, 14 cmH2O** | | | |
| --- | --- | --- | --- |
| **L0** | **IS0** | **ISIE** | **P value** |
| **Trigger delay [ms]** | 80 [80 - 80] | 80 [70 - 80] | 0.315 |
| **Inspiratory time in excess [s]** | 0.44 [0.44 – 0.46] | 0.24 [0.23 – 0.24] | <0.001 |
| **PTP_vent_ trigger [cmH_2_O*s]** | 0.260 [0.246 - 0.267] | 0.178 [0.173 - 0.196] | <0.001 |
| **PTP_vent_300 [cmH_2_O*s]** | 0.923 [0.866 - 0.959] | 1.072 [0.974 - 1.145] | 0.011 |
| **PTP_vent_ 500 [cmH_2_O*s]** | 3.162 [3.135 - 3.183] | 3.36 [3.192 - 3.438] | 0.035 |
| **Classical asynchronies** |  | | |
| - **Auto-triggering [%]** | 0 | 0 | 1.000 |
| - **Early cycling [%]** | 0 | 0 | 1.000 |
| - **Delayed cycling [%]** | 0 | 0 | 1.000 |
| **L9** | **IS0** | **ISIE** | **P value** |
| **Trigger delay [ms]** | 80 [80 - 80] | 80 [60 - 80] | 0,105 |
| **Inspiratory time in excess [s]** | 0.42 [0.33 – 0.51] | 0.21 [0.18 – 0.24] | <0.001 |
| **PTP_vent_ trigger [cmH_2_O*s]** | 0.194 [0.184 - 0.198] | 0.145 [0.130 - 0.149] | <0.001 |
| **PTP_vent_300 [cmH_2_O*s]** | 0.842 [0.783 - 0.917] | 0.900 [0.791 - 1.002] | 0.218 |
| **PTP_vent_ 500 [cmH_2_O*s]** | 3.005 [2.967 - 3.143] | 3.088 [3.002 - 3.218] | 0.190 |
| **Classical asynchronies** |  | | |
| - **Auto-triggering [%]** | 0 | 0 | 1.000 |
| - **Early cycling [%]** | 0 | 0 | 1.000 |
| - **Delayed cycling [%]** | 0 | 0 | 1.000 |
| **L20** | **IS0** | **ISIE** | **P value** |
| **Trigger delay [ms]** | 70 [60 - 80] | 60 [60 - 80] | 0,481 |
| **Inspiratory time in excess [s]** | 0.61 [0.56 – 0.64] | 0.16 [0.07 – 0.18] | <0.001 |
| **PTP_vent_ trigger [cmH_2_O*s]** | 0.145 [0.140 - 0.159] | 0.097 [0.088 - 0.102] | <0.001 |
| **PTP_vent_300 [cmH_2_O*s]** | 0.720 [0.667 - 0.767] | 0.773 [0.734 - 0.821] | 0.063 |
| **PTP_vent_ 500 [cmH_2_O*s]** | 2.717 [2.624 - 2.745] | 2.809 [2.766 - 2.859] | 0.029 |
| **Classical asynchronies** |  | | |
| - **Auto-triggering [%]** | 0 | 0 | 1.000 |
| - **Early cycling [%]** | 0 | 0 | 1.000 |
| - **Delayed cycling [%]** | 0 | 0 | 1.000 |

**eTable 6**: Results at steady state for obstructive ventilatory mechanics and an pressure support of 14cmH_2_O. L0 : no leak; L9 : leak flow of 9L/min; L20 : leak flow of 20L/min. IS0 : IntelliSync+® deactivated; ISIE : IntelliSync+® activated during inspiratory and expiratory phase.

| **Restrictive respiratory mechanics, 8 cmH2O** | | | |
| --- | --- | --- | --- |
| **L0** | **IS0** | **ISIE** | **P value** |
| **Trigger delay [ms]** | 120 [100 - 120] | 100 [100 - 100] | 0.315 |
| **Inspiratory time in excess [s]** | 0.03 [0.03 – 0.04] | 0.03 [0.03 – 0.06] | 0.796 |
| **PTP_vent_ trigger [cmH_2_O*s]** | 0.479 [0.472 - 0.493] | 0.512 [0.504 - 0.519] | 0.002 |
| **PTP_vent_300 [cmH_2_O*s]** | -0.234 [-0.272 - -0.197] | -0.193 [-0.212 - -0.182] | 0.247 |
| **PTP_vent_ 500 [cmH_2_O*s]** | 1.050 [0.975 - 1.104] | 1.162 [1.104 - 1.231] | 0.023 |
| **Classical asynchronies** |  | | |
| - **Auto-triggering [%]** | 0 | 0 | 1.000 |
| - **Early cycling [%]** | 0 | 0 | 1.000 |
| - **Delayed cycling [%]** | 0 | 0 | 1.000 |
| **L9** | **IS0** | **ISIE** | **P value** |
| **Trigger delay [ms]** | NA | 90 [80 - 100] | NA |
| **Inspiratory time in excess [s]** | 0.08 [0.08 – 0.10] | 0.04 [0.04 – 0.06] | 0.002 |
| **PTP_vent_ trigger [cmH_2_O*s]** | NA | 0.333 [0.315 - 0.342] | NA |
| **PTP_vent_300 [cmH_2_O*s]** | NA | -0.094 [-0.133 - -0.086] | NA |
| **PTP_vent_ 500 [cmH_2_O*s]** | NA | 0.999 [0.921 - 1.027] | NA |
| **Classical asynchronies** |  | | |
| - **Auto-triggering [%]** | 35 | 0 | 0.333 |
| - **Early cycling [%]** | 0 | 0 | 1.000 |
| - **Delayed cycling [%]** | 0 | 0 | 1.000 |
| **L20** | **IS0** | **ISIE** | **P value** |
| **Trigger delay [ms]** | NA | NA | NA |
| **Inspiratory time in excess [s]** | 0.10 [0.10 – 0.16] | -0.02 [-0.02 – 0.02] | <0.001 |
| **PTP_vent_ trigger [cmH_2_O*s]** | NA | NA | NA |
| **PTP_vent_300 [cmH_2_O*s]** | NA | NA | NA |
| **PTP_vent_ 500 [cmH_2_O*s]** | NA | NA | NA |
| **Classical asynchronies** |  | | |
| - **Auto-triggering [%]** | 85 | 33 | 0.333 |
| - **Early cycling [%]** | 0 | 0 | 1.000 |
| - **Delayed cycling [%]** | 0 | 0 | 1.000 |

**eTable 7**: Results at steady state for restrictive ventilatory mechanics and an pressure support of 8cmH_2_O. L0 : no leak; L9 : leak flow of 9L/min; L20 : leak flow of 20L/min. IS0 : IntelliSync+® deactivated; ISIE : IntelliSync+® activated during inspiratory and expiratory phase.

| **Restrictive respiratory mechanics, 14 cmH2O** | | | |
| --- | --- | --- | --- |
| **L0** | **IS0** | **ISIE** | **P value** |
| **Trigger delay [ms]** | 80 [80 - 100] | 100 [100 - 100] | 0,063 |
| **Inspiratory time in excess [s]** | 0.28 [0.28 – 0.32] | 0.22 [0.22 – 0.30] | 0.247 |
| **PTP_vent_ trigger [cmH_2_O*s]** | 0.431 [0.418 - 0.439] | 0.443 [0.433 - 0.453] | 0.035 |
| **PTP_vent_300 [cmH_2_O*s]** | 0.305 [0.241 - 0.329] | 0.256 [0.251 - 0.275] | 0.315 |
| **PTP_vent_ 500 [cmH_2_O*s]** | 2.243 [2.175 - 2.347] | 2.284 [2.230 - 2.299] | 0.912 |
| **Classical asynchronies** |  | | |
| - **Auto-triggering [%]** | 0 | 0 | 1.000 |
| - **Early cycling [%]** | 0 | 0 | 1.000 |
| - **Delayed cycling [%]** | 0 | 0 | 1.000 |
| **L9** | **IS0** | **ISIE** | **P value** |
| **Trigger delay [ms]** | 80 [80 - 80] | 80 [80 - 80] | 0.481 |
| **Inspiratory time in excess [s]** | 0.30 [0.30 – 0.32] | 0.22 [0.22 – 0.26] | <0.001 |
| **PTP_vent_ trigger [cmH_2_O*s]** | 0.344 [0.329 - 0.352] | 0.264 [0.257 - 0.271] | <0.001 |
| **PTP_vent_300 [cmH_2_O*s]** | 0.440 [0.388 - 0.455] | 0.531 [0.472 - 0.568] | 0.004 |
| **PTP_vent_ 500 [cmH_2_O*s]** | 2.469 [2.397 - 2.550] | 2.641 [2.493 - 2.690] | 0.029 |
| **Classical asynchronies** |  | | |
| - **Auto-triggering [%]** | 15 | 0 | 0.667 |
| - **Early cycling [%]** | 0 | 0 | 1.000 |
| - **Delayed cycling [%]** | 0 | 0 | 1.000 |
| **L20** | **IS0** | **ISIE** | **P value** |
| **Trigger delay [ms]** | 60 [60 - 80] | 50 [40 - 60] | 0.011 |
| **Inspiratory time in excess [s]** | 0.33 [0.33 – 0.40] | 0.21 [0.21 – 0.26] | <0.001 |
| **PTP_vent_ trigger [cmH_2_O*s]** | 0.274 [0.251 - 0.291] | 0.185 [0.179 - 0.188] | <0.001 |
| **PTP_vent_300 [cmH_2_O*s]** | 0.424 [0.388 - 0.507]] | 0.543 [0.503 - 0.588] | 0.007 |
| **PTP_vent_ 500 [cmH_2_O*s]** | 2.419 [2.377 - 2.495] | 2.488 [2.407 - 2.609] | 0.123 |
| **Classical asynchronies** |  | | |
| - **Auto-triggering [%]** | 0 | 0 | 1.000 |
| - **Early cycling [%]** | 0 | 0 | 1.000 |
| - **Delayed cycling [%]** | 5 | 0 | 0.667 |

**eTable 8**: Results at steady state for restrictive ventilatory mechanics and an pressure support of 14cmH_2_O. L0 : no leak; L9 : leak flow of 9L/min; L20 : leak flow of 20L/min. IS0 : IntelliSync+® deactivated; ISIE : IntelliSync+® activated during inspiratory and expiratory phase.

**eFigure 4** shows the auto-triggering asynchrony index at steady state for the different set-ups evaluated.


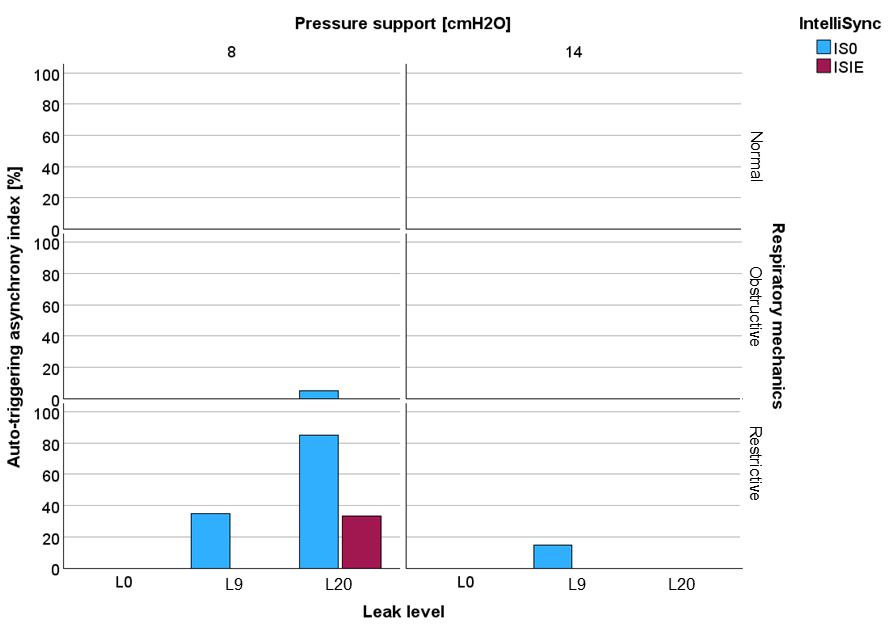


**eFigure 4**: Asynchrony index for auto-triggering at steady state, for all the tested set-ups.
IS0: IntelliSync+® deactivated; ISI: IntelliSync+® activated during inspiration phase; ISE: IntelliSync+® activated during expiratory phase; ISIE: IntelliSync+® activated during inspiratory and expiratory phase.

**eFigure 5** and **eFigure 6** respectively illustrate the PTP_vent_trig and PTP_vent_300 values obtained for all tested conditions.


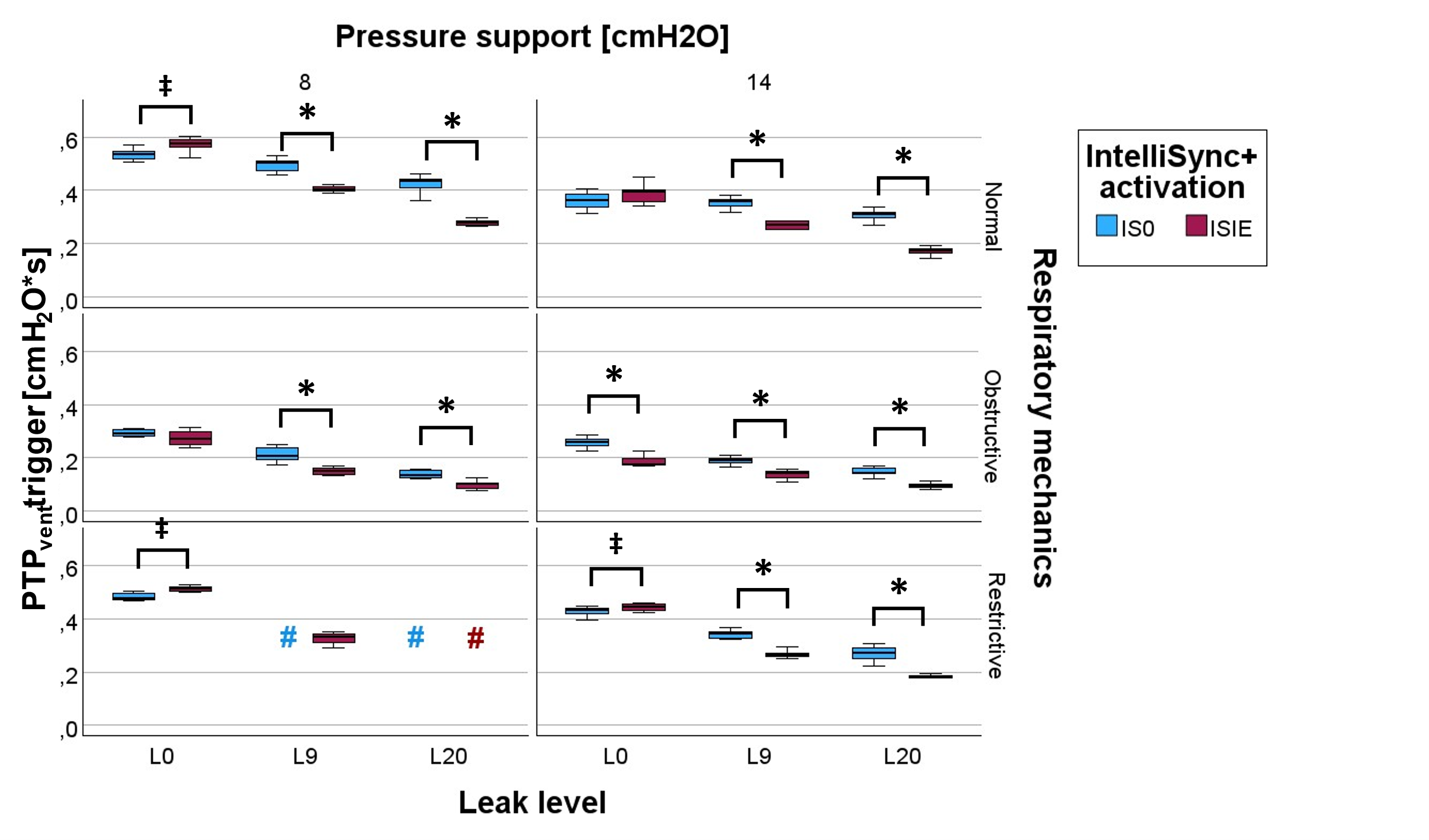


**eFigure 5**: Triggering ventilatory pressure-time product (PTP_vent_trig) in the various tested set-ups. *p < 0.05, in favor of ISIE. ‡p < 0.05 in favor of IS0. L0: no leak flow; L9: leak flow of 9L/min; L20: leak flow of 20L/min. IS0: IntelliSync+® deactivated; ISIE: IntelliSync+® activated during inspiratory and expiratory phase. #Unstable set-up, with no PTP_vent_trig measurement possible.


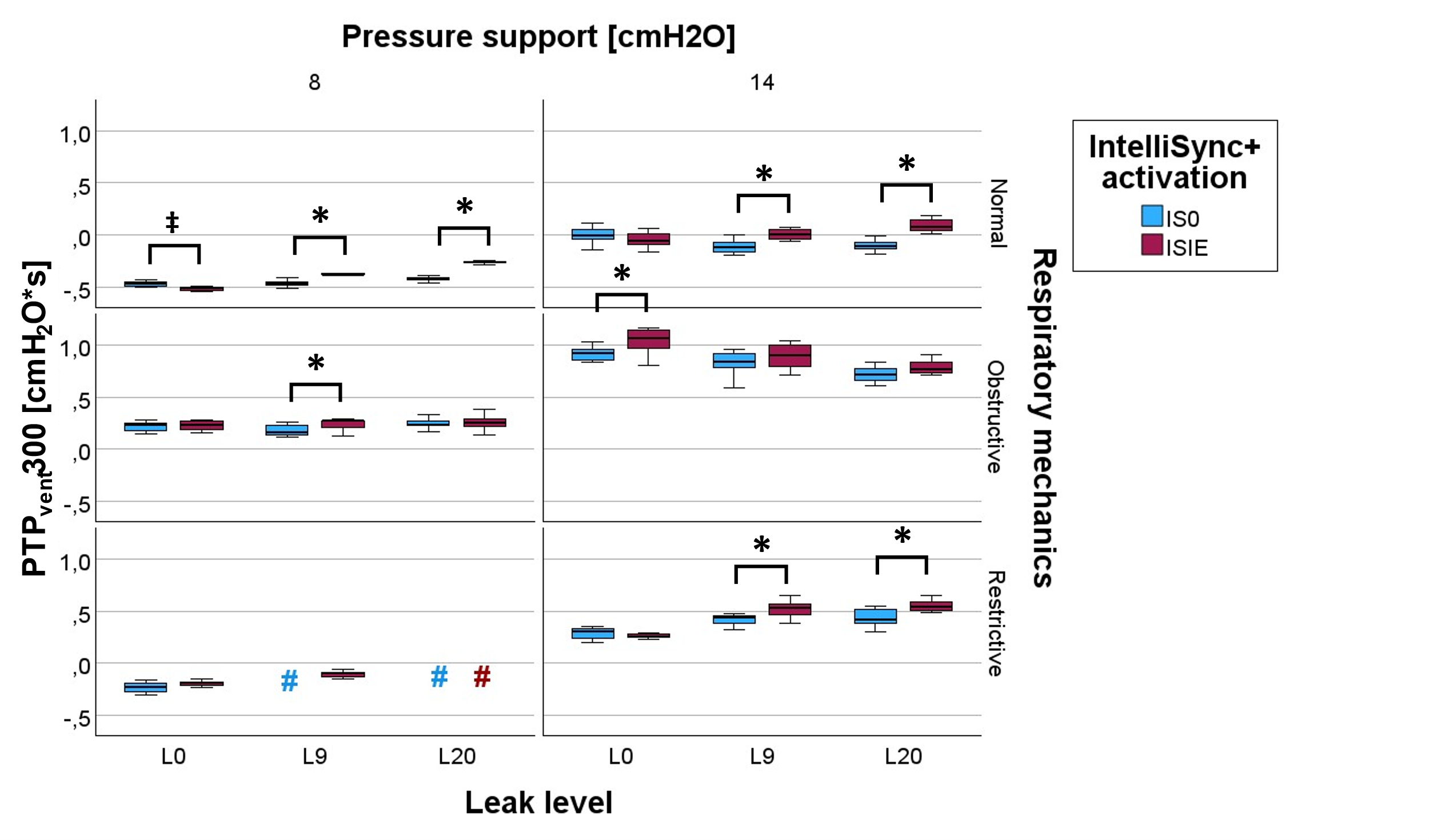


**eFigure 6**: PTP_vent_300 in the various tested set-ups. *p < 0.05, in favor of ISIE. ‡p < 0.05, in favor of IS0. L0: no leak flow; L9: leak flow of 9L/min; L20: leak flow of 20L/min. IS0: IntelliSync+® deactivated; ISIE: IntelliSync+® activated during inspiratory and expiratory phase. # Unstable set-up, with no PTP_vent_300 measurement possible.

- 1. *Major asynchronies IS0 vs ISIE Post-modification*

***eFigure 7*** illustrates the auto-triggering asynchronies directly after increasing the leak level from 0 L/min to 20 L/min with and without IntelliSync+^®^ activation. Of note, no statistically significant result was obtained.


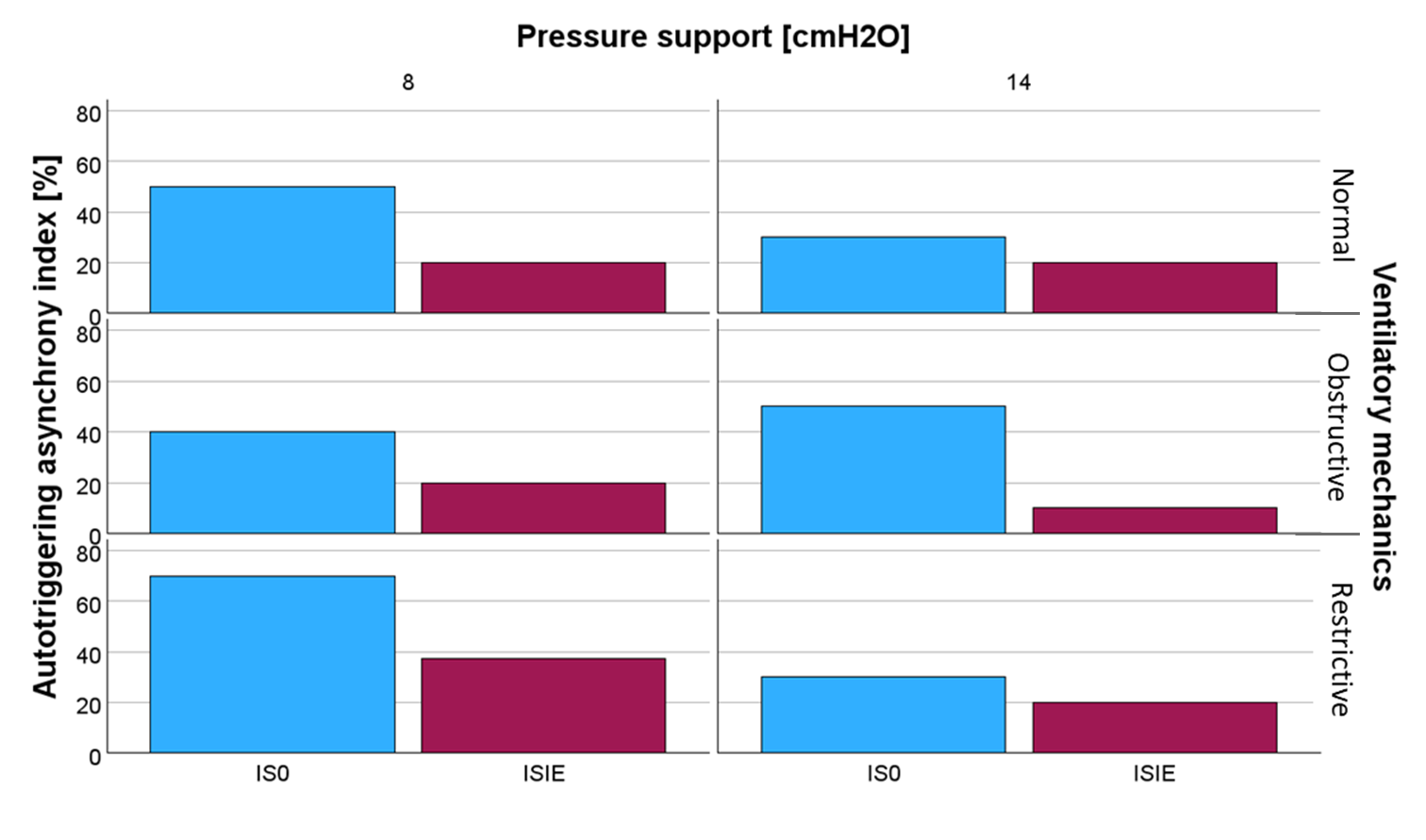


**eFigure 7:** Auto-triggering asynchrony indexes immediately after leak increase from L0 (0 L/min) to L20 (20 L/min).
For all conditions, the occurrence of auto-triggering was counted during the first 10 respiratory cycles of the driver ventilator directly following leak increase. Of note, no statistical significance could be demonstrated on these results. IS0: IntelliSync+® deactivated; ISIE: IntelliSync+® activated during inspiratory and expiratory phase.

***eTable 9*** presents the results regarding classical asynchronies post leak increase.

|  | | | **IS0** | | **ISIE** | |
| --- | --- | --- | --- | --- | --- | --- |
|  |  |  | **AT** | **LC** | **AT** | **LC** |
| **Normal** | **8 cmH_2_O** | **L0 to L9** | **20** | 0 | **40** | 0 |
|  |  | **L9 to L20** | **20** | 0 | **50** | 0 |
|  |  | **L0 to L20** | **50** | 0 | **20** | 0 |
|  | **14cmH_2_O** | **L0 to L9** | **20** | 0 | **10** | 0 |
|  |  | **L9 to L20** | **20** | 0 | 0 | 0 |
|  |  | **L0 to L20** | **30** | 0 | **20** | 0 |
| **Obstructive** | **8 cmH_2_O** | **L0 to L9** | **30** | 0 | **30** | 0 |
|  |  | **L9 to L20** | **40** | 0 | **40** | 0 |
|  |  | **L0 to L20** | **40** | 0 | **20** | 0 |
|  | **14cmH_2_O** | **L0 to L9** | **10** | 0 | **20** | 0 |
|  |  | **L9 to L20** | **10** | 0 | **20** | 0 |
|  |  | **L0 to L20** | **50** | 0 | **10** | 0 |
| **Restrictive** | **8 cmH_2_O** | **L0 to L9** | **30** | 0 | **10** | 0 |
|  |  | **L9 to L20** | **80** | 0 | **41** | 0 |
|  |  | **L0 to L20** | **70** | **10** | **38** | 0 |
|  | **14cmH_2_O** | **L0 to L9** | **30** | 0 | **10** | 0 |
|  |  | **L9 to L20** | **30** | **10** | **20** | 0 |
|  |  | **L0 to L20** | **30** | **60** | **20** | 0 |

**eTable 9** : Classical asynchronies after leak increase. Asynchrony indexes (expressed in %) are mentioned for each setp-up.
IS0: IntelliSync+® deactivated; ISIE: IntelliSync+® activated during inspiratory and expiratory phase. L0: no leak; L9 : leak flow of 9L/min; L20 : leak flow of 20L/min. AT : auto-triggering, LC : late cycling. Green cells: asynchrony index 0. Yellow cells: asynchrony index >0 and <50%. Orange cells: asynchrony index > 50%.

- 1. *ISI vs ISIE and ISE vs ISIE at steady state*

***eTable 10*** *to* ***eTable 15*** present the results obtained during our study, at steady state. The indicated p-value refers to a comparison between ISI, respectively ISE when applicable, and ISIE.

| **Normal mechanics, pressure support 8 cmH_2_O** | | | | |
| --- | --- | --- | --- | --- |
| **L0** | **ISIE** | **ISI** | **ISE** | **P value** |
| **Trigger delay [ms]** | 120 [120 - 120] | 120 [120 - 120] | NR | 0.739 |
| **Inspiratory time in excess [s]** | 0.05 [0.04 – 0.06] | NR | 0.06 [0.05 – 0.06] | 0.353 |
| **PTP_vent_ trigger [cmH_2_O*s]** | 0.575 [0.561 - 0.588] | 0.601 [0.553 - 0.614] | NR | 0.218 |
| **PTP_vent_300 [cmH_2_O*s]** | -0.513 [-0.531 - -0.503] | -0.544 [-0.549 - -0.489] | NR | 0.247 |
| **PTP_vent_ 500 [cmH_2_O*s]** | 0.132 [0.074 - 0.146] | 0.135 [0.091 - 0.188] | NR | 0.796 |
| **Classical asynchronies** |  | | | |
| - **Auto-triggering [%]** | 0 | 0 | NR | 1.000 |
| - **Early cycling [%]** | 0 | NR | 0 | 1.000 |
| - **Delayed cycling [%]** | 0 | NR | 0 | 1.000 |
| **L9** | **ISIE** | **ISI** | **ISE** | **P value** |
| **Trigger delay [ms]** | 120 [100 - 120] | 100 [100 - 100] | NR | 0.165 |
| **Inspiratory time in excess [s]** | 0.06 [0.06 – 0.12] | NR | 0.06 [0.06 – 0.12] | 0.971 |
| **PTP_vent_ trigger [cmH_2_O*s)** | 0.403 [0.397 - 0.411] | 0.413 [0.389 - 0.429] | NR | 0.529 |
| **PTP_vent_300 [cmH_2_O*s)** | -0.384 [-0.385 - -0.375] | -0.386 [-0.420 - -0.365] | NR | 0.631 |
| **PTP_vent_ 500 [cmH_2_O*s)** | 0.076 [0.014 - 0.100] | 0.05 [-0.041 - 0.084] | NR | 0.190 |
| **Classical asynchronies** |  | | | |
| - **Auto-triggering [%]** | 0 | 0 | NR | 1.000 |
| - **Early cycling [%]** | 0 | NR | 0 | 1.000 |
| - **Delayed cycling [%]** | 0 | NR | 0 | 1.000 |
| **L20** | **ISIE** | **ISI** | **ISE** | **P value** |
| **Trigger delay [ms]** | 60 [60 - 60] | 60 [60 - 80] | NR | 0.280 |
| **Inspiratory time in excess [s]** | 0.07 [0.06 – 0.08] | NR | 0.08 [0.07 – 0.14] | 0.190 |
| **PTP_vent_ trigger [cmH_2_O*s]** | 0.278 [0.272 - 0.285]* | 0.307 [0.296 - 0.314]* | NR | <0.001 |
| **PTP_vent_300 [cmH_2_O*s]** | -0.267 [-0.272 - -0.259]* | -0.295 [-0.308 - -0.286]* | NR | <0.001 |
| **PTP_vent_ 500 [cmH_2_O*s]** | 0.089 [0.047 - 0.101] | 0.079 [0.022 - 0.085] | NR | 0.218 |
| **Classical asynchronies** |  | | | |
| - **Auto-triggering [%]** | 0 | 0 | NR | 1.000 |
| - **Early cycling [%]** | 0 | NR | 0 | 1.000 |
| - **Delayed cycling [%]** | 0 | NR | 0 | 1.000 |

**eTable 10:** Results at steady state for a normal ventilatory mechanics and an pressure support of 8cmH_2_O. For partial activation of IntelliSync+®, only relevant results are shown (NR = not relevant). P-value calculated between partial and full IntelliSync+® activation, with p < 0.05 significance (*). L0 : no leak; L9 : leak flow of 9L/min; L20 : leak flow of 20L/min. IS0 : IntelliSync+® deactivated; ISI : IntelliSync+® activated during inspiratory phase; ISE : IntelliSync+® activated during expiratory phase; ISIE : IntelliSync+® activated during inspiratory and expiratory phase.

| **Normal mechanics, pressure support 14 cmH_2_O** | | | | |
| --- | --- | --- | --- | --- |
| **L0** | **ISIE** | **ISI** | **ISE** | **P value** |
| **Trigger delay [ms]** | 100 [100 - 100] | 100 [100 - 100] | NR | 1.000 |
| **Inspiratory time in excess [s]** | 0.16 [0.16 – 0.20] | NR | 0.16 [0.16 – 0.18] | 0.684 |
| **PTP_vent_ trigger [cmH_2_O*s]** | 0.396 [0.365 - 0.399] | 0.387 [0.382 - 0.395] | NR | 0.684 |
| **PTP_vent_300 [cmH_2_O*s]** | -0.059 [-0.093 - 0.004] | -0.075 [-0.086 - -0.012] | NR | 0.912 |
| **PTP_vent_ 500 [cmH_2_O*s]** | 1.485 [1.432 - 1.583] | 1.396 [1.328 - 1.507] | NR | 0.247 |
| **Classical asynchronies** |  | | | |
| - **Auto-triggering [%]** | 0 | 0 | NR | 1.000 |
| - **Early cycling [%]** | 0 | NR | 0 | 1.000 |
| - **Delayed cycling [%]** | 0 | NR | 0 | 1.000 |
| **L9** | **ISIE** | **ISI** | **ISE** | **P value** |
| **Trigger delay [ms]** | 100 [100 - 120] | 100 [100 - 100] | NR | 0.796 |
| **Inspiratory time in excess [s]** | 0.20 [0.19 – 0.22]* | NR | 0.22 [0.20 – 0.24]* | 0.043 |
| **PTP_vent_ trigger [cmH_2_O*s]** | 0.273 [0.257 - 0.282] | 0.278 [0.268 - 0.285] | NR | 0.218 |
| **PTP_vent_300 [cmH_2_O*s]** | 0.010 [-0.033 - 0.043] | -0.020 [-0.048 - 0.005] | NR | 0.315 |
| **PTP_vent_ 500 [cmH_2_O*s]** | 1.319 [1.270 - 1.375] | 1.245 [1.183 - 1.340] | NR | 0.165 |
| **Classical asynchronies** |  | | | |
| - **Auto-triggering [%]** | 0 | 0 | NR | 1.000 |
| - **Early cycling [%]** | 0 | NR | 0 | 1.000 |
| - **Delayed cycling [%]** | 0 | NR | 0 | 1.000 |
| **L20** | **ISIE** | **ISI** | **ISE** | **P value** |
| **Trigger delay [ms]** | 60 [60 - 60] | 60 [60 - 80] | NR | 0.393 |
| **Inspiratory time in excess [s]** | 0.21 [0.20 – 0.26] | NR | 0.20 [0.19 – 0.24] | 0.280 |
| **PTP_vent_ trigger [cmH_2_O*s]** | 0.175 [0.167 - 0.180] | 0.181 [0.169 - 0.191] | NR | 0.218 |
| **PTP_vent_300 [cmH_2_O*s]** | 0.073 [0.049 - 0.130] | 0.052 [0.035 - 0.083] | NR | 0.353 |
| **PTP_vent_ 500 [cmH_2_O*s]** | 1.232 [1.191 - 1.339] | 1.208 [1.055 - 1.318] | NR | 0.393 |
| **Classical asynchronies** |  | | | |
| - **Auto-triggering [%]** | 0 | 0 | NR | 1.000 |
| - **Early cycling [%]** | 0 | NR | 0 | 1.000 |
| - **Delayed cycling [%]** | 0 | NR | 0 | 1.000 |

**eTable 11**: Results at steady state for normal ventilatory mechanics and an pressure support of 14cmH_2_O. For partial activation of IntelliSync+®, only relevant results are shown (NR = not relevant). P-value calculated between partial and full IntelliSync+® activation, with p < 0.05 significance (*). L0 : no leak; L9 : leak flow of 9L/min; L20 : leak flow of 20L/min. IS0 : IntelliSync+® deactivated; ISI : IntelliSync+® activated during inspiratory phase; ISE : IntelliSync+® activated during expiratory phase; ISIE : IntelliSync+® activated during inspiratory and expiratory phase.

| **Obstructive mechanics, pressure support 8 cmH_2_O** | | | | |
| --- | --- | --- | --- | --- |
| **L0** | **ISIE** | **ISI** | **ISE** | **P value** |
| **Trigger delay [ms]** | 100 [90 - 100] | 90 [80 - 100] | NR | 0.481 |
| **Inspiratory time in excess [s]** | 0.22 [0.21 – 0.22] | NR | 0.22 [0.22 – 0.22] | 0.971 |
| **PTP_vent_ trigger [cmH_2_O*s]** | 0.271 [0.248 - 0.298] | 0.297 [0.273 - 0.301] | NR | 0.393 |
| **PTP_vent_300 [cmH_2_O*s]** | 0.238 [0.186 - 0.272] | 0.207 [0.185 - 0.234] | NR | 0.529 |
| **PTP_vent_ 500 [cmH_2_O*s]** | 1.403 [1.399 - 1.444] | 1.399 [1.385 - 1.420] | NR | 0.436 |
| **Classical asynchronies** |  | | | |
| - **Auto-triggering [%]** | 0 | 0 | NR | 1.000 |
| - **Early cycling [%]** | 0 | NR | 0 | 1.000 |
| - **Delayed cycling [%]** | 0 | NR | 0 | 1.000 |
| **L9** | **ISIE** | **ISI** | **ISE** | **P value** |
| **Trigger delay [ms]** | 60 [60 - 80] | 60 [60 - 60] | NR | 0.529 |
| **Inspiratory time in excess [s]** | 0.27 [0.23 – 0.28] | NR | 0.22 [0.21 – 0.26] | 0.105 |
| **PTP_vent_ trigger [cmH_2_O*s]** | 0.153 [0.139 - 0.162] | 0.145 [0.142 - 0.154] | NR | 0.631 |
| **PTP_vent_300 [cmH_2_O*s]** | 0.272 [0.213 - 0.272] | 0.244 [0.230 - 0.264] | NR | 1.000 |
| **PTP_vent_ 500 [cmH_2_O*s]** | 1.358 [1.281 - 1.376] | 1.322 [1.291 - 1.357] | NR | 0.739 |
| **Classical asynchronies** |  | | | |
| - **Auto-triggering [%]** | 0 | 0 | NR | 1.000 |
| - **Early cycling [%]** | 0 | NR | 0 | 1.000 |
| - **Delayed cycling [%]** | 0 | NR | 0 | 1.000 |
| **L20** | **ISIE** | **ISI** | **ISE** | **P value** |
| **Trigger delay [ms]** | 60 [60 - 80] | 60 [60 - 60] | NR | 0.247 |
| **Inspiratory time in excess [s]** | 0.03 [-0.01 – 0.09] | NR | 0.06 [0.01 – 0.08] | 0.631 |
| **PTP_vent_ trigger [cmH_2_O*s]** | 0.102 [0.088 - 0.106] | 0.087 [0.084 - 0.091] | NR | 0.105 |
| **PTP_vent_300 [cmH_2_O*s]** | 0.257 [0.225 - 0.285] | 0.290 [0.254 - 0.319] | NR | 0.190 |
| **PTP_vent_ 500 [cmH_2_O*s]** | 1.223 [1.140 - 1.272] | 1.243 [1.185 - 1.311] | NR | 0.436 |
| **Classical asynchronies** |  | | | |
| - **Auto-triggering [%]** | 0 | 0 | NR | 1.000 |
| - **Early cycling [%]** | 0 | NR | 0 | 1.000 |
| - **Delayed cycling [%]** | 0 | NR | 0 | 1.000 |

**eTable 12**: Results at steady state for obstructive ventilatory mechanics and an pressure support of 8cmH_2_O. For partial activation of IntelliSync+®, only relevant results are shown (NR = not relevant). P-value calculated between partial and full IntelliSync+® activation, with p < 0.05 significance (*). L0 : no leak; L9 : leak flow of 9L/min; L20 : leak flow of 20L/min. IS0 : IntelliSync+® deactivated; ISI : IntelliSync+® activated during inspiratory phase; ISE : IntelliSync+® activated during expiratory phase; ISIE : IntelliSync+® activated during inspiratory and expiratory phase.

| **Obstructive mechanics, pressure support 14 cmH_2_O** | | | | |
| --- | --- | --- | --- | --- |
| **L0** | **ISIE** | **ISI** | **ISE** | **P value** |
| **Trigger delay [ms]** | 80 [70 - 80] | 80 [80 - 100] | NR | 0.247 |
| **Inspiratory time in excess [s]** | 0.24 [0.23 – 0.24] | NR | 0.22 [0.22 – 0.22] | 0.052 |
| **PTP_vent_ trigger [cmH_2_O*s]** | 0.178 [0.173 - 0.196] | 0.201 [0.182 - 0.212] | NR | 0.105 |
| **PTP_vent_300 [cmH_2_O*s]** | 1.072 [0.974 - 1.145] | 1.009 [0.900 - 1.026] | NR | 0.315 |
| **PTP_vent_ 500 [cmH_2_O*s]** | 3.36 [3.192 - 3.438] | 3.263 [3.233 - 3.332] | NR | 0.529 |
| **Classical asynchronies** |  | | | |
| - **Auto-triggering [%]** | 0 | 0 | NR | 1.000 |
| - **Early cycling [%]** | 0 | NR | 0 | 1.000 |
| - **Delayed cycling [%]** | 0 | NR | 0 | 1.000 |
| **L9** | **ISIE** | **ISI** | **ISE** | **P value** |
| **Trigger delay [ms]** | 80 [60 - 80] | 60 [60 - 80] | NR | 0.315 |
| **Inspiratory time in excess [s]** | 0.21 [0.18 – 0.24] | NR | 0.19 [0.16 – 0.26] | 1.000 |
| **PTP_vent_ trigger [cmH_2_O*s]** | 0.145 [0.130 - 0.149] | 0.139 [0.133 - 0.143] | NR | 0.393 |
| **PTP_vent_300 [cmH_2_O*s]** | 0.900 [0.791 - 1.002] | 0.853 [0.830 - 0.904] | NR | 0.853 |
| **PTP_vent_ 500 [cmH_2_O*s]** | 3.088 [3.002 - 3.218] | 3.085 [2.980 - 3.143] | NR | 0.631 |
| **Classical asynchronies** |  | | | |
| - **Auto-triggering [%]** | 0 | 0 | NR | 1.000 |
| - **Early cycling [%]** | 0 | NR | 0 | 1.000 |
| - **Delayed cycling [%]** | 0 | NR | 0 | 1.000 |
| **L20** | **ISIE** | **ISI** | **ISE** | **P value** |
| **Trigger delay [ms]** | 60 [60 - 80] | 60 [60 - 60] | NR | 0.739 |
| **Inspiratory time in excess [s]** | 0.16 [0.07 – 0.18] | NR | 0.14 [0.11 – 0.20] | 0.853 |
| **PTP_vent_ trigger [cmH_2_O*s]** | 0.097 [0.088 - 0.102] | 0.090 [0.088 - 0.094] | NR | 0.218 |
| **PTP_vent_300 [cmH_2_O*s]** | 0.773 [0.734 - 0.821] | 0.883 [0.833 - 0.902] | NR | 0.123 |
| **PTP_vent_ 500 [cmH_2_O*s]** | 2.809 [2.766 - 2.859] | 2.933 [2.856 - 2.959] | NR | 0.075 |
| **Classical asynchronies** |  | | | |
| - **Auto-triggering [%]** | 0 | 0 | NR | 1.000 |
| - **Early cycling [%]** | 0 | NR | 0 | 1.000 |
| - **Delayed cycling [%]** | 0 | NR | 0 | 1.000 |

**eTable 13**: Results at steady state for obstructive ventilatory mechanics and an pressure support of 14cmH_2_O. For partial activation of IntelliSync+®, only relevant results are shown (NR = not relevant). P-value calculated between partial and full IntelliSync+® activation, with p < 0.05 significance (*). L0 : no leak; L9 : leak flow of 9L/min; L20 : leak flow of 20L/min. IS0 : IntelliSync+® deactivated; ISI : IntelliSync+® activated during inspiratory phase; ISE : IntelliSync+® activated during expiratory phase; ISIE : IntelliSync+® activated during inspiratory and expiratory phase.

| **Restrictive mechanics, pressure support 8 cmH_2_O** | | | | |
| --- | --- | --- | --- | --- |
| **L0** | **ISIE** | **ISI** | **ISE** | **P value** |
| **Trigger delay [ms]** | 100 [100 - 100] | 100 [100 - 120] | NR | 0.579 |
| **Inspiratory time in excess [s]** | 0.03 [0.03 – 0.06] | NR | 0.03 [0.03 – 0.04] | 0.436 |
| **PTP_vent_ trigger [cmH_2_O*s]** | 0.512 [0.504 - 0.519] | 0.510 [0.497 - 0.517] | NR | 0.684 |
| **PTP_vent_300 [cmH_2_O*s]** | -0.193 [-0.212 - -0.182] | -0.209 [-0.246 - -0.198] | NR | 0.315 |
| **PTP_vent_ 500 [cmH_2_O*s]** | 1.162 [1.104 - 1.231] | 1.139 [1.097 - 1.178] | NR | 0.353 |
| **Classical asynchronies** |  | | | |
| - **Auto-triggering [%]** | 0 | 0 | NR | 1.000 |
| - **Early cycling [%]** | 0 | NR | 0 | 1.000 |
| - **Delayed cycling [%]** | 0 | NR | 0 | 1.000 |
| **L9** | **ISIE** | **ISI** | **ISE** | **P value** |
| **Trigger delay [ms]** | 90 [80 - 100] | 100 [100 - 100] | NR | 0.143 |
| **Inspiratory time in excess [s]** | 0.04 [0.04 – 0.06] | NR | 0.02 [0.02 – 0.04] | 0.247 |
| **PTP_vent_ trigger [cmH_2_O*s]** | 0.333 [0.315 - 0.342] | 0.308 [0.297 - 0.343] | NR | 0.393 |
| **PTP_vent_300 [cmH_2_O*s]** | -0.094 [-0.133 - -0.086] | -0.119 [-0.180 - -0.084] | NR | 0.579 |
| **PTP_vent_ 500 [cmH_2_O*s]** | 0.999 [0.921 - 1.027] | 0.960 [0.886 - 1.002] | NR | 0.280 |
| **Classical asynchronies** |  | | | |
| - **Auto-triggering [%]** | 0 | 0 | NR | 1.000 |
| - **Early cycling [%]** | 0 | NR | 0 | 1.000 |
| - **Delayed cycling [%]** | 0 | NR | 0 | 1.000 |
| **L20** | **ISIE** | **ISI** | **ISE** | **P value** |
| **Trigger delay [ms]** | NA | NA | NR | NA |
| **Inspiratory time in excess [s]** | -0.02 [-0.02 – 0.02] | NR | 0.01 [0.01 – 0.02] | 0.280 |
| **PTP_vent_ trigger [cmH_2_O*s]** | NA | NA | NR | NA |
| **PTP_vent_300 [cmH_2_O*s]** | NA | NA | NR | NA |
| **PTP_vent_ 500 [cmH_2_O*s]** | NA | NA | NR | NA |
| **Classical asynchronies** |  | | | |
| - **Auto-triggering [%]** | 33 | 46 | NR | 1.000 |
| - **Early cycling [%]** | 0 | NR | 0 | 1.000 |
| - **Delayed cycling [%]** | 0 | NR | 0 | 1.000 |

**eTable 14**: Results at steady state for restrictive ventilatory mechanics and an pressure support of 8cmH_2_O For partial activation of IntelliSync+®, only relevant results are shown (NR = not relevant). P-value calculated between partial and full IntelliSync+® activation, with p < 0.05 significance (*). L0 : no leak; L9 : leak flow of 9L/min; L20 : leak flow of 20L/min. IS0 : IntelliSync+® deactivated; ISI : IntelliSync+® activated during inspiratory phase; ISE : IntelliSync+® activated during expiratory phase; ISIE : IntelliSync+® activated during inspiratory and expiratory phase.

| **Restrictive mechanics, pressure support 14 cmH_2_O** | | | | |
| --- | --- | --- | --- | --- |
| **L0** | **ISIE** | **ISI** | **ISE** | **P value** |
| **Trigger delay [ms]** | 100 [100 - 100]* | 80 [80 - 100]* | NR | 0.023 |
| **Inspiratory time in excess [s]** | 0.22 [0.22 – 0.30] | NR | 0.25 [0.25 – 0.28] | 0.0.853 |
| **PTP_vent_ trigger [cmH_2_O*s]** | 0.443 [0.433 - 0.453] | 0.440 [0.429 - 0.442] | NR | 0.481 |
| **PTP_vent_300 [cmH_2_O*s]** | 0.256 [0.251 - 0.275] | 0.289 [0.267 - 0.312] | NR | 0.063 |
| **PTP_vent_ 500 [cmH_2_O*s]** | 2.284 [2.230 - 2.299] | 2.271 [2.263 - 2.293] | NR | 0.684 |
| **Classical asynchronies** |  | | | |
| - **Auto-triggering [%]** | 0 | 0 | NR | 1.000 |
| - **Early cycling [%]** | 0 | NR | 0 | 1.000 |
| - **Delayed cycling [%]** | 0 | NR | 0 | 1.000 |
| **L9** | **ISIE** | **ISI** | **ISE** | **P value** |
| **Trigger delay [ms]** | 80 [80 - 80] | 80 [80 - 100] | NR | 0.684 |
| **Inspiratory time in excess [s]** | 0.22 [0.22 – 0.26] | NR | 0.20 [0.20 – 0.22] | 0.052 |
| **PTP_vent_ trigger [cmH_2_O*s]** | 0.264 [0.257 - 0.271]* | 0.285 [0.284 - 0.290]* | NR | 0.015 |
| **PTP_vent_300 [cmH_2_O*s]** | 0.531 [0.472 - 0.568] | 0.493 [0.425 - 0.520] | NR | 0.315 |
| **PTP_vent_ 500 [cmH_2_O*s]** | 2.641 [2.493 - 2.690] | 2.610 [2.502 - 2.627] | NR | 0.436 |
| **Classical asynchronies** |  | | | |
| - **Auto-triggering [%]** | 0 | 0 | NR | 1.000 |
| - **Early cycling [%]** | 0 | NR | 0 | 1.000 |
| - **Delayed cycling [%]** | 0 | NR | 0 | 1.000 |
| **L20** | **ISIE** | **ISI** | **ISE** | **P value** |
| **Trigger delay [ms]** | 50 [40 - 60] | 50 [40 - 60] | NR | 1.000 |
| **Inspiratory time in excess [s]** | 0.21 [0.21 – 0.26] | NR | 0.21 [0.21 – 0.28] | 0.739 |
| **PTP_vent_ trigger [cmH_2_O*s]** | 0.185 [0.179 - 0.188] | 0.182 [0.173 - 0.188] | NR | 0.796 |
| **PTP_vent_300 [cmH_2_O*s]** | 0.543 [0.503 - 0.588] | 0.564 [0.545 - 0.590] | NR | 0.796 |
| **PTP_vent_ 500 [cmH_2_O*s]** | 2.488 [2.407 - 2.609] | 2.542 [2.490 - 2.597] | NR | 0.739 |
| **Classical asynchronies** |  | | | |
| - **Auto-triggering [%]** | 0 | 0 | NR | 1.000 |
| - **Early cycling [%]** | 0 | NR | 0 | 1.000 |
| - **Delayed cycling [%]** | 0 | NR | 0 | 1.000 |

**eTable 15**: Results at steady state for restrictive ventilatory mechanics and an pressure support of 14cmH_2_O. For partial activation of IntelliSync+®, only relevant results are shown (NR = not relevant). P-value calculated between partial and full IntelliSync+® activation, with p < 0.05 significance (*). L0 : no leak; L9 : leak flow of 9L/min; L20 : leak flow of 20L/min. IS0 : IntelliSync+® deactivated; ISI : IntelliSync+® activated during inspiratory phase; ISE : IntelliSync+® activated during expiratory phase; ISIE : IntelliSync+® activated during inspiratory and expiratory phase.

1. Bibliography

1. Sheehy RD, Duce B, Edwards TP, Churton JA, Sharma R, Hukins CA. Double-Triggering During Noninvasive Ventilation in a Simulated Lung Model. Respir Care. 2020 Sep;65(9):1333–8.

2. Miyoshi E, Fujino Y, Uchiyama A, Mashimo T, Nishimura M. Effects of Gas Leak on Triggering Function, Humidification, and Inspiratory Oxygen Fraction During Noninvasive Positive Airway Pressure Ventilation. Chest. 2005 Nov;128(5):3691–8.

3. Beloncle F, Piquilloud L, Olivier PY, Vuillermoz A, Yvin E, Mercat A, et al. Accuracy of P0.1 measurements performed by ICU ventilators: a bench study. Ann Intensive Care. 2019 Dec;9(1):104.

4. Ferreira JC, Chipman DW, Hill NS, Kacmarek RM. Bilevel vs ICU Ventilators Providing Noninvasive Ventilation: Effect of System Leaks. Chest. 2009 Aug;136(2):448–56.

5. Oto J, Chenelle CT, Marchese AD, Kacmarek RM. A Comparison of Leak Compensation in Acute Care Ventilators During Noninvasive and Invasive Ventilation: A Lung Model Study. Respiratory Care. 2013 Dec 1;58(12):2027–37.

6. Olivieri C, Costa R, Conti G, Navalesi P. Bench studies evaluating devices for non-invasive ventilation: critical analysis and future perspectives. Intensive Care Med. 2012 Jan;38(1):160–7.
